# EZH2-mediated epigenetic silencing of TIMP2 promotes ovarian cancer migration and invasion

**DOI:** 10.1038/s41598-017-03362-z

**Published:** 2017-06-15

**Authors:** Xiaoqing Yi, Jianfeng Guo, Jing Guo, Si Sun, Ping Yang, Junjie Wang, Yuan Li, Lisha Xie, Jing Cai, Zehua Wang

**Affiliations:** 1 Department of Obstetrics and Gynecology, Union Hospital, Tongji Medical College, Huazhong University of Science and Technology, Wuhan, 430022 China; 2 Department of Obstetrics and Gynecology, First Affiliated Hospital, School of Medicine, Shihezi University, Shihezi, 832008 China; 3 Department of Obstetrics and Gynecology, Renhe Hospital, Three Gorges University, Yichang, 443001 China

## Abstract

Enhancer of zeste homolog 2 (EZH2) is often increased in malignant tumors and is involved in metastasis. EZH2 silences gene expression by tri-methylating the lysine 27 residue of histone H3 (H3K27me3). However, the mechanism underlying EZH2 promotion of ovarian cancer metastasis remains elusive. Here, we showed that EZH2 is up-regulated in ovarian cancer and is associated with tumor metastasis and poor survival by mRNA sequencing and microarray results from databases. Tissue microarray and immunohistochemistry results revealed that EZH2 was negatively correlated with the expression of tissue inhibitor of metalloproteinases 2 (TIMP2). EZH2 overexpression inhibited TIMP2 expression and promoted proteolytic activities of matrix metalloproteinases 2 and 9 and vice versa. EZH2 promoted ovarian cancer invasion and migration, which could be largely reversed by TIMP2 down-regulation *in vitro* and *in vivo*. Both H3K27me3 inhibition and demethylation could reduce methylation of the TIMP2 promoter and finally reactivate TIMP2 transcription. The presence of EZH2 and H3K27me3 at the TIMP2 promoter was confirmed by chromatin immunoprecipitation. H3K27me3 and DNA methyltransferases at the promoter were significantly increased by EZH2 overexpression. These results suggest that EZH2 inhibits TIMP2 expression via H3K27me3 and DNA methylation, which relieve the repression of MMP and facilitate ovarian cancer invasion and migration.

## Introduction

Ovarian cancer is the most lethal gynecological malignancy. In 2015, it was estimated that 52,100 Chinese women were newly diagnosed with ovarian cancer and approximately 22,500 women died of this disease^1^. Approximately 61% of ovarian cancer patients have distant metastasis at diagnosis; the five-year survival rate for patients with distant metastasis is less than 30%, whereas the survival rate in patients without distant metastasis can be as high as 92%^2^. Given that metastasis is a strong independent risk factor for poor survival in ovarian cancer, it is extremely important to elucidate the underlying molecular mechanisms, which may improve the precise molecular diagnosis and lead to new treatments for ovarian cancer.

Enhancer of zeste homolog 2 (EZH2) is the enzymatic subunit of polycomb repressive complex 2 (PRC2) that catalyzes the tri-methylation of histone H3 lysine 27 (H3K27me3) to induce chromatin compaction and subsequent transcriptional silencing^3, 4^. In addition, EZH2 can lead to promoter hypermethylation-mediated silencing by recruiting DNA methyltransferases to the CpG-rich promoter regions of target genes^5^. EZH2 is highly expressed in a wide range of cancer types and has been implicated in the progression and metastasis of hepatocellular carcinoma, bladder cancer and melanoma^6–8^. EZH2 plays a vital role in tumorigenesis and tumor progression due to its canonical function as an epigenetic silencer^9^, and the development and clinical application of EZH2-specific inhibitors have been an active area of investigation. EZH2 inhibitors have shown promising results in preclinical studies, suggesting they may have clinical applications^3^. Our previous work showed that EZH2 is up-regulated in epithelial ovarian cancer (EOC) and that high EZH2 expression significantly correlates with poor cell differentiation, advanced FIGO (The International Federation of Gynecology and Obstetrics) stage and positive lymph node metastasis^10^. Moreover, the depletion of EZH2 inhibited ovarian cancer progression *in vitro* and *in vivo* ^10, 11^. These findings indicate that EZH2 is a key driver of an aggressive phenotype of ovarian cancer, prompting us to investigate the mechanism underlying the effects of EZH2. This study may contribute to the application of EZH2 inhibitors in ovarian cancer therapy.

Matrix metalloproteinases (MMPs) facilitate tumor cell invasion by degrading a variety of extracellular matrix (ECM) components, which is one of the key mechanisms of metastasis initiation^12^. More than 20 MMPs have been identified to date. Among these MMPs, MMP2 and MMP9 (also known as gelatinase A and B), which degrade type IV collagens in ECM, have been implicated in ovarian cancer^13^. *Schmalfeldt B et al*. reported dramatic increases in MMP2, MMP9, and active MMP2 in advanced ovarian cancers and omentum metastases compared with benign ovarian tumors and tumors with low malignant potential^14^. Moreover, high MMP2 and MMP9 expression promotes ovarian cancer cell invasion and is associated with tumor progression and poor survival in ovarian cancer patients^15, 16^. Tissue inhibitors of metalloproteinases (TIMPs) are endogenous regulators of MMPs that inhibit MMP activity by binding to the active sites of MMPs to form 1:1 stoichiometric complexes or sequestering the pro-MMP zymogens^17^. Four TIMPs have been identified: TIMP1, TIMP2, TIMP3, and TIMP4. The four TIMPs can inhibit all 23 human MMPs with varied inhibitory spectrums and activities^17^. TIMPs are down-regulated in many solid tumors and act as repressors of tumor metastasis^18–21^. *Yang*, *S*. *W*. *et al*. delivered ectopic TIMP2 to the mouse abdominal cavity using conditionally replicating adenovirus expressing TIMP2 in the mouse model. This upregulation of TIMP2 delayed tumor growth and significantly increased survival^20^, indicating that TIMP regulation may have applications in cancer treatment. However, the regulation of TIMP expression in ovarian cancer remains largely unclear.

In the present study, bioinformatics analysis of RNA sequencing microarray data confirmed the high EZH2 expression in ovarian cancer and its correlation with metastasis and poor patient survival. In addition, the correlations between TIMP and EZH2 expression levels suggested that TIMP2 was a potential target of EZH2. To verify the regulation of TIMP2 by EZH2, we performed loss- and gain-of-function experiments of EZH2 and examined TIMP2 expression, MMP2 and MMP9 expression and proteolytic activity, and tumor cell migration and invasion. Moreover, the contribution of TIMP2 to the functions of EZH2 was assessed *in vivo*, and we demonstrated that the epigenetic modifications on the TIMP2 promoter are mediated by EZH2, inhibiting TIMP2 expression.

## Results

### High EZH2 expression confers metastasis and poor patient survival in ovarian cancer

Analysis of EZH2 mRNA expression data from TCGA database and GEO portal revealed that EZH2 was significantly up-regulated in ovarian cancer compared with normal controls (Supplemental Fig. S1a–d) and high EZH2 expression was related to advanced FIGO stage, poor differentiation, and metastasis (Table 1). Moreover, the overall survival time in patients with high EZH2 expression was significantly shorter than those with low EZH2 expression (Supplemental Fig. S1e–h). These findings indicated that EZH2 promotes ovarian cancer progression and metastasis.

**Table 1:** Correlation of EZH2 expression with clinicopathological status in ovarian cancer patients.

Clinicopathological status	*N*	EZH2^b^		*P* value^c^
		High (%)	Low (%)	
**GSE9891**				
**Age** (**year**)				0.883
>50	232	174 (75)	58 (25)	
≤50	50	37 (74.0)	13 (26.0)	
Unknown^a^	3	2	1	
**FIGO stage**				**0**.**001**
I	24	12 (50.0)	12 (50.0)	
II	18	12 (66.7)	6 (33.3)	
III	217	175 (80.6)	42 (19.4)	
IV	22	12 (54.5)	10 (45.5)	
Unknown^a^	4	2	2	
**Histologic grade**				**7**.**8276e**-**7**
G1	19	5 (26.3)	14 (73.7)	
G2	97	71 (73.2)	26 (26.8)	
G3	164	134 (81.7)	30 (18.3)	
Unknown^a^	5	3	2	
**Histological type**				0.27
Serous	264	195 (73.9)	69 (26.1)	
Endometrioid	20	17 (85.0)	3 (15.0)	
Unknown^a^	1	1	0	
**Metastasis**				**0**.**003**
Yes	257	199 (77.4)	58 (22.7)	
No	24	12 (50.0)	12 (50.0)	
Unknown^a^	4	2	2	
**GSE26193**				
**FIGO stage**				**0**.**004** ^**d**^
I	20	9 (45.0)	11 (55.0)	
II	11	8 (72.7)	3 (27.3)	
III	59	47 (79.7)	12 (20.3)	
IV	17	14	3	
**Histological grade**				**0**.**004**
G1	7	2 (28.6)	5 (71.4)	
G2	33	21 (63.6)	12 (36.4)	
G3	67	55 (82.1)	12 (17.9)	
**Histological type**				0.485
Serous	79	59 (74.7)	20 (25.3)	
Others^e^	28	19 (67.9)	9 (32.1)	
**Metastasis**				**0**.**002**
Yes	87	69 (79.3)	18 (20.7)	
No	20	9 (45.0)	11 (55.0)	
Unknown^a^	4	2	2	
**The present study**				
**Age** (**year**)				0.647
>50	41	25 (61.0)	16 (39.0)	
≤50	41	27 (65.9)	14 (34.1)	
**FIGO stage**				**0**.**002**
I-II	22	8 (36.4)	14 (63.6)	
III-IV	60	44 (73.3)	16 (26.7)	
**Histological type** ^**f**^				0.152
Type I	20	10 (50.0)	10 (50.0)	
Type II	62	42 (67.7)	20 (32.3)	

^a^Statistics did not include the Unknown group.

^b^Cut-off point for EZH2 mRNA from databases was determined by ROC curve. Cut-off point for EZH2 protein was defined as tissues whose positive staining nuclear percentage were greater than 25%.

^c^Chi-square test.

^d^Fisher’s exact tests.

^e^Include mucinous, endometrioid, clear cells, carcinosarcome and brenner tumor.

^f^Type I includes low-grade serous, endometrioid, mucinous, and clear cell tumors; Type II includes high-grade serous tumor.

Boldface type indicates significant values.

### EZH2 inversely correlates with TIMP2 expression in ovarian cancer

**Figure 1:**
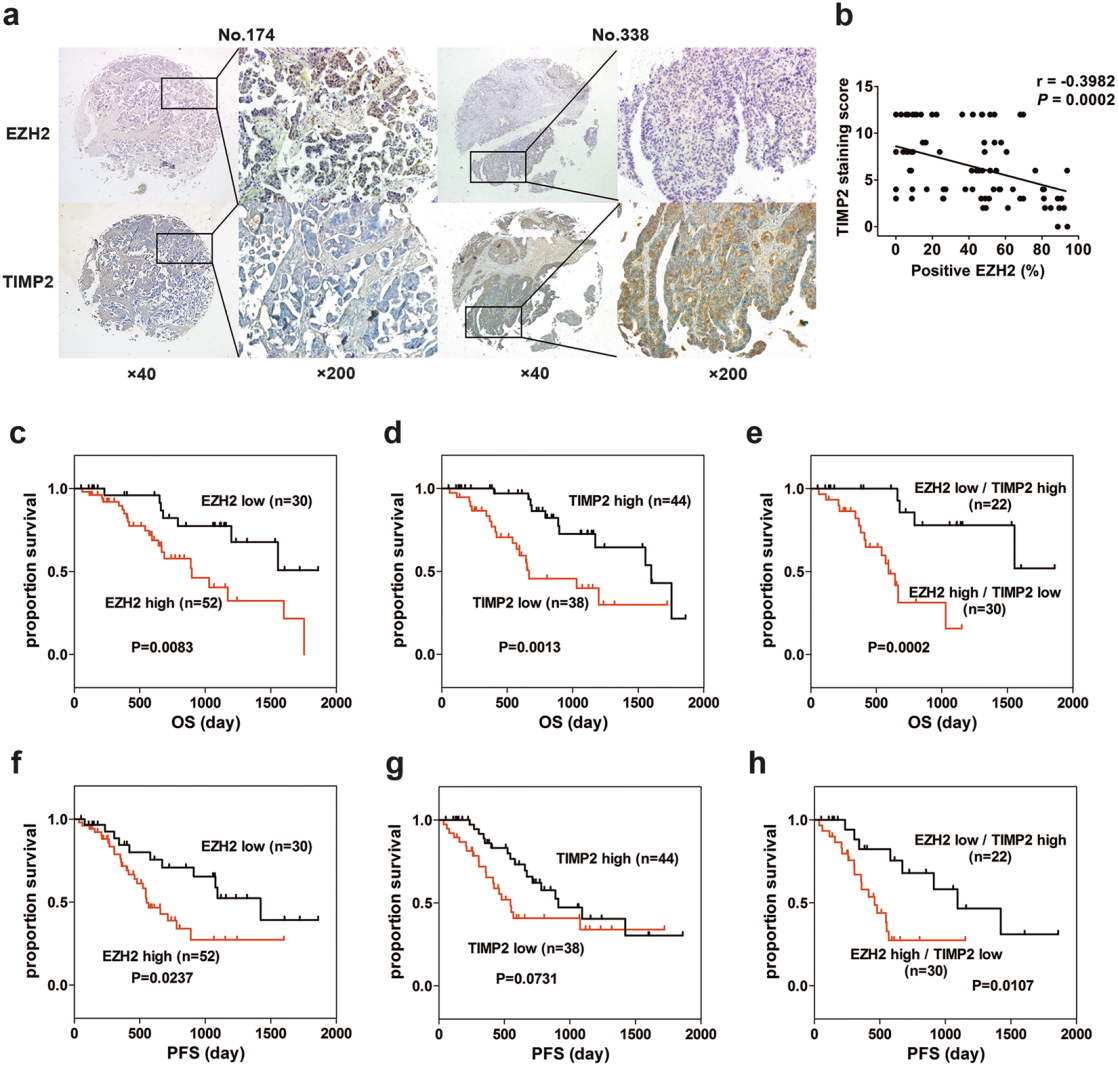
EZH2 inversely correlates with TIMP2 and indicates poor prognosis. (**a**) Representative images of immunohistochemistry analysis of EZH2 and TIMP2 in ovarian cancer tissues. EZH2 was expressed in the nucleus, while TIMP2 was expressed in the cytoplasm. Sample No. 174 has high EZH2 expression and low TIMP2 expression. Sample No. 338 has low EZH2 expression and high TIMP2 expression (scale bar, 50 μm). (**b**) Negative correlation between the percentage of EZH2-positive cells and the TIMP2 staining score in ovarian cancer tissues (Spearman’s correlation test). (**c**–**e**) Kaplan-Meier plots for overall survival in epithelial ovarian cancer patients. High EZH2 (**c**), low TIMP2 (**d**) or high EZH2 and simultaneous low TIMP2 (**e**) are associated with reduced overall survival. (**f**–**h**) Kaplan-Meier plots for progression-free survival in patients with epithelial ovarian cancer stratified by the expression status of EZH2 (**f**), TIMP2 (**g**), or both EZH2 and TIMP2 (**h**).

To identify potential targets of EZH2 in TIMP family, the relationship among EZH2 expression and TIMPs was analyzed. Based on the mRNA expression data in GSE9891, GSE26193 and GSE49997, EZH2 was negatively associated with the expression of TIMP2 but not TIMP1, TIMP3 or TIMP4 (Supplemental Fig. S2). Given the strong inhibition of MMPs by TIMP2, TIMP2 was chosen as the potential target gene of EZH2. To verify the relationship between EZH2 and TIMP2, their expression levels were examined in 82 EOC tissues using a tissue microarray combined with IHC. As illustrated in Fig. 1a, EZH2 was predominantly expressed in the nuclei, while TIMP2 was located in the cytoplasm. Semi-quantitative analysis of IHC results revealed a significant inverse correlation between EZH2 and TIMP2 expression (Fig. 1b). Furthermore, Kaplan-Meier survival analysis showed that high EZH2 expression and low TIMP2 expression were significantly associated with reduced overall survival and progression-free survival in patients with EOC (Fig. 1c–g).

### EZH2 inhibits TIMP2 expression

**Figure 2:**
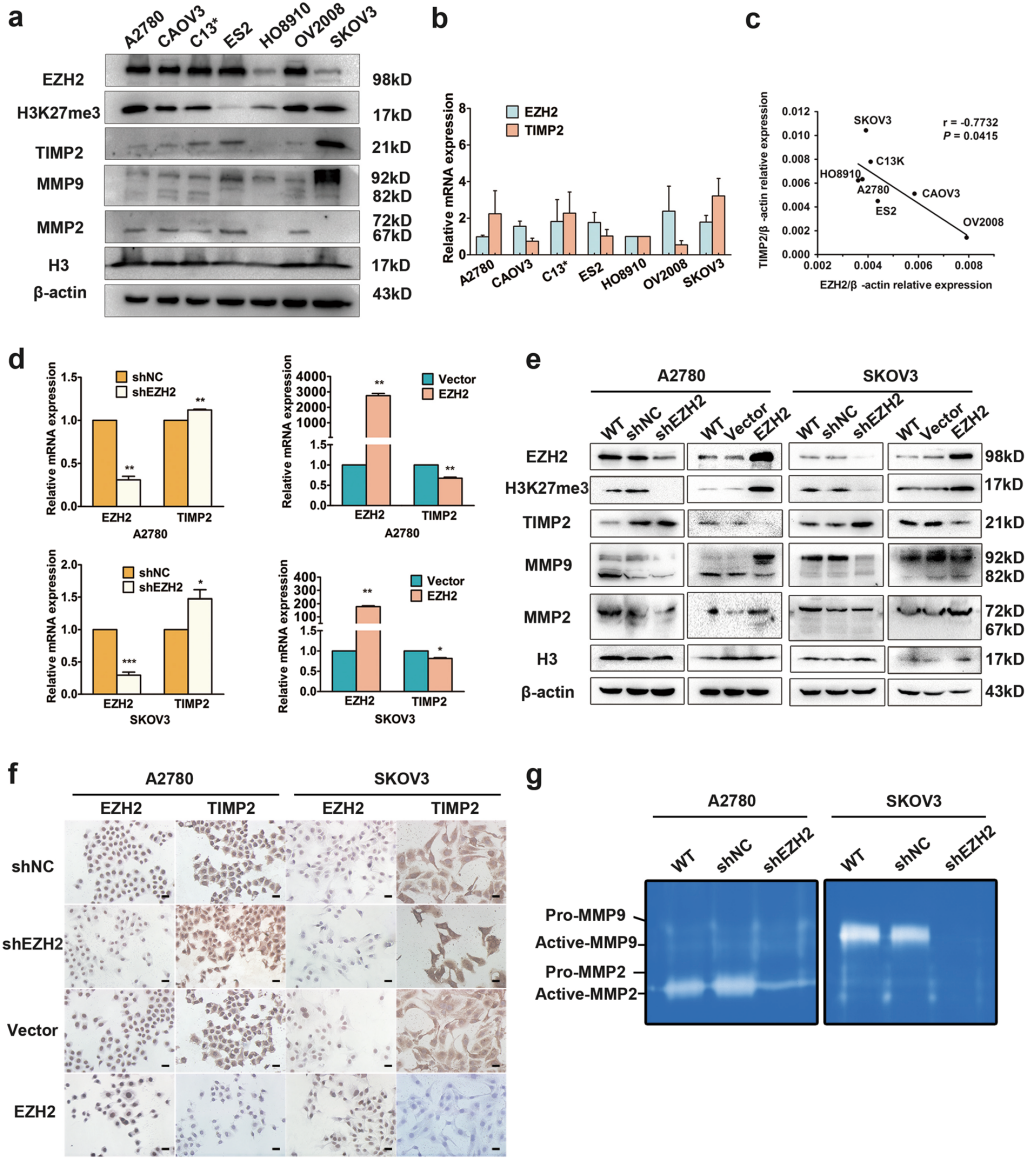
EZH2 regulates TIMP2 expression *in vitro*. (**a**) Western blot analysis of EZH2, TIMP2, MMP2, and MMP9 protein expression in ovarian cancer cell lines. Histone-3 (H3) and β-actin serve as internal controls. The cropped blots are used in the figure, and full-length blots are presented in Supplementary Fig. S5. (**b**) EZH2 and TIMP2 mRNA expression levels in seven ovarian cancer cell lines were detected by quantitative real-time PCR. (**c**) Negative correlation between TIMP2 and EZH2 mRNA levels in ovarian cancer cell lines. (**d**) qRT-PCR for EZH2 and TIMP2 expression in A2780 and SKOV3 cells with EZH2 up- or down-regulation by transfection with an EZH2-overexpressing plasmid (EZH2) or targeting shRNA (shEZH2). (**e**) Western blot analysis of EZH2, TIMP2 and MMP2/9 protein expression in EZH2-regulated A2780 and SKOV3 cells. Histone-3 (H3) and β-actin are used as internal controls. The cropped blots are used in the figure, and full-length blots are presented in Supplementary Fig. S6. (**f**) Immunocytochemical staining of EZH2 and TIMP2 in EZH2-regulated A2780 and SKOV3 cells (scale bar, 20 μm). (**g**) Gelatin zymography assays of MMP2 and MMP9 activity in EZH2-regulated A2780 and SKOV3 cells. The cropped gels are used in the figure, and full-length gels are presented in Supplementary Fig. S7. *P < 0.05; **P < 0.01; ***P < 0.001.

To further investigate whether EZH2 regulates TIMP2 expression, western blot analysis and RT-PCR were performed in seven epithelial ovarian cancer cell lines. High EZH2 and low TIMP2 protein levels were found in A2780, CAOV3, C13*, ES2, and OV2008, while low EZH2 and high TIMP2 were observed in SKOV3 (Fig. 2a). In addition, there was a strong negative correlation between EZH2 and TIMP2 mRNA expression levels in these cell lines (Fig. 2b,c). The RT-PCR, western blot and immunocytochemistry results showed that ectopic overexpression of EZH2 inhibited the expression of TIMP2 and *vice versa* (Fig. 2d–f). In addition, EZH2 negatively regulated MMP2 and MMP9 expression and enzyme activity (Fig. 2e,g). These findings suggested that EZH2 inhibits TIMP2 and MMPs.

### EZH2 promotes ovarian cancer invasion and migration by repressing TIMP2 *in vitro*

**Figure 3:**
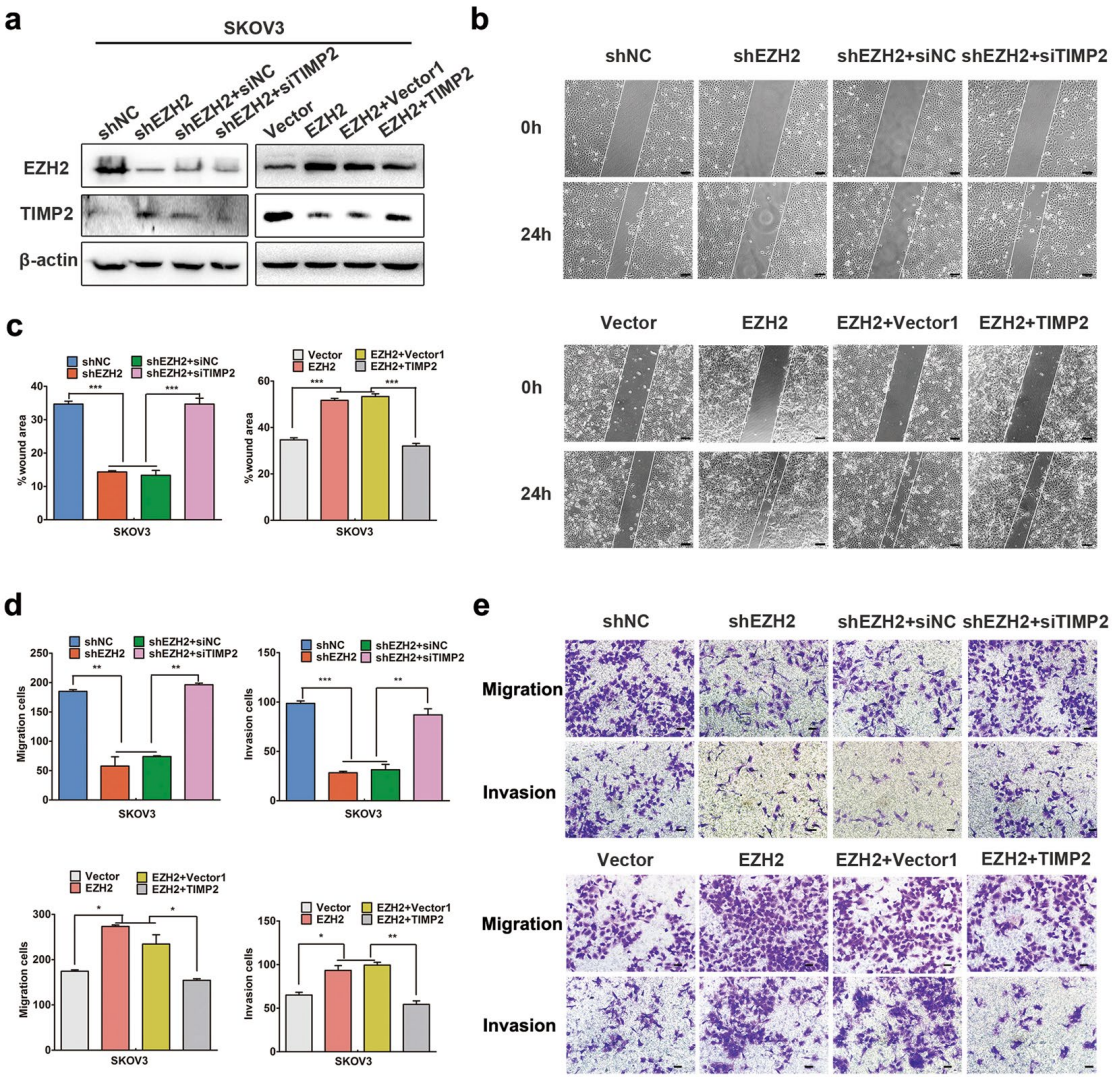
EZH2 promotes ovarian cancer cell invasion and migration by repressing TIMP2. Cells were transfected with shEZH2, shEZH2 and siTIMP2; EZH2-plasmid, EZH2-plasmid and TIMP2-plasmid and corresponding controls. (**a**) Western blot analysis of EZH2 and TIMP2 in SKOV3 cells after co-transfection. The cropped blots are used in the figure, and full-length blots are presented in Supplementary Fig. S8. (**b**) Scratch-wound assays for the detection of cell mobility (scale bar, 100 μm). (**c**) Histograms show the wound-healing areas of the scratch-wound assays. (**d**) Transwell migration and invasion assays. The number of invading and migrating cells is presented. (**e**) Representative images of migration and invasion assays (scale bar, 50 μm). *P < 0.05; **P < 0.01; ***P < 0.001.

To detect the effects of EZH2-mediated TIMP2 repression on ovarian cancer cell migration and invasion, scratch-wound assays and transwell migration and invasion assays were performed. As shown in Fig. 3 and Supplemental Fig. S3, we found that depletion of EZH2 resulted in significant decreases in migration and invasion of A2780 and SKOV3 cells. However, simultaneous treatment with TIMP2 siRNA partially reversed the EZH2 knockdown-induced reduction in cell migration and invasion. Similarly, the enhanced migration and invasion of ovarian cancer cells induced by ectopic EZH2 expression could be relieved by co-expression of TIMP2. These results indicated that repression of TIMP2 is required for the EZH2-mediated increase in migration and invasion of ovarian cancer cells.

### EZH2 promotes ovarian cancer metastasis by repressing TIMP2 *in vivo*

**Figure 4:**
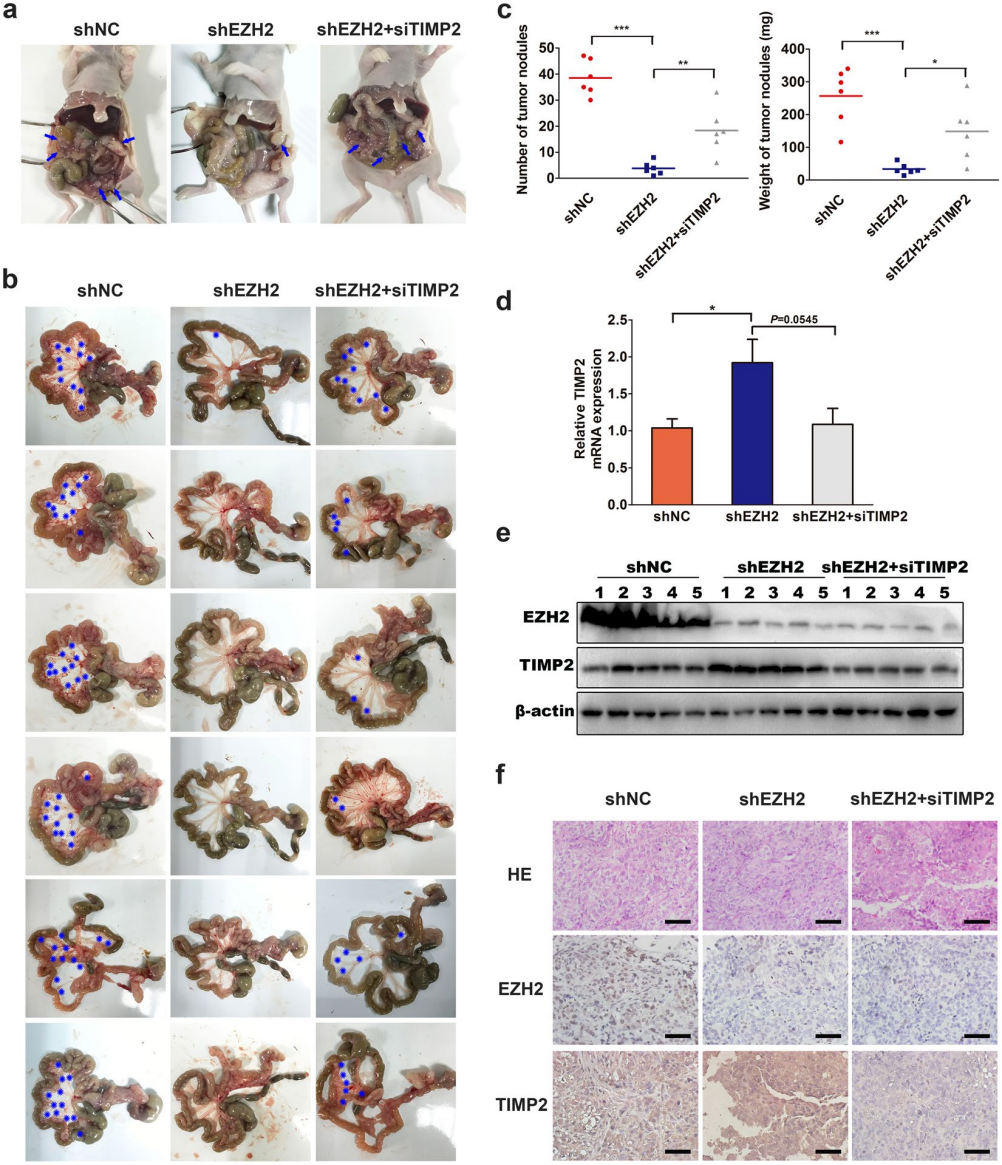
Down-regulation of EZH2 inhibits ovarian cancer metastasis by elevating TIMP2 in xenograft models. Nude mice were intraperitoneally inoculated with SKOV3 cells stably transfected with shNC or shEZH2. *In vitro* transient transfection and *in vivo* transfection of siTIMP2 was performed in one subgroup inoculated with SKOV3-shEZH2. (**a**) Peritoneal dissemination of xenografts. SKOV3-shEZH2 cells formed many fewer i.p. metastatic nodules (blue arrow) in the abdominal cavity of mice than SKOV3-shNC cells. In contrast, the transfection of siTIMP2 could significantly increase the reduced tumor metastatic nodules mediated by EZH2 depletion. (**b**) Representative images of the mesenteric membrane of six mice per group are shown. *Indicates tumor nodule. (**c**) The histogram of quantified weight of metastatic nodules >8 mm^3^ and the number of metastatic nodules in three groups at the time of sacrifice are shown. (**d**) RT-PCR results of TIMP2 mRNA expression of the eighteen SKOV3 xenografts in three groups. (**e**) Western blot analysis of EZH2 and TIMP2 protein expression of the xenografts. Five tissues for each group are shown because one tissue in the shEZH2 group and one in the cotransfected group were too small for protein extraction. The cropped blots are used in the figure, and full-length blots are presented in Supplementary Fig. S9. (**f**) The hematoxylin and eosin staining and immunohistochemical analysis of xenografts. EZH2 and TIMP2 protein expressions in control and intervention groups were verified. (scale bar, 20 μm). * *P* < 0.05; **P < 0.01; ***P < 0.001.

To investigate the role of the EZH2-TIMP2 axis in ovarian cancer metastasis *in vivo*, we established a disseminated ovarian cancer model in BALB/c nude mice by intraperitoneal injection of tumor cells. The ovarian cancer xenografts were derived from SKOV3 cells that were stably transfected with shNC or shEZH2. Three days before tumor cell injection, half of the SKOV3-shEZH2 cells were transiently transfected with TIMP2 siRNA (co-transfection group). SKOV3-shNC cells, SKOV3-shEZH2 cell or co-transfected cells were intraperitoneally injected (i.p.). *In vivo* TIMP2 siRNA delivery was conducted once a week. The SKOV3-shEZH2 cells generated dramatically fewer and smaller tumor nodules (>8 mm^3^) in the peritoneal cavity than the SKOV3-shNC cells. Correspondingly, compared to the control group, the weight of tumor nodules was reduced approximately 90% in the SKOV3-shEZH2 group. In the co-transfection group, the number and the size of the tumor nodules were significantly increased compared to the SKOV3-shEZH2 group but not fully restored to the extent in the control group (Fig. 4a–c). The expression levels of EZH2 and TIMP2 in xenografts were validated by RT-PCR, western blot and immunohistochemistry (Fig. 4d–f). These findings indicated that EZH2 promotes ovarian cancer metastasis partly by repressing the expression of TIMP2.

### EZH2 inhibits TIMP2 expression via DNA methylation and H3K27me3

**Figure 5:**
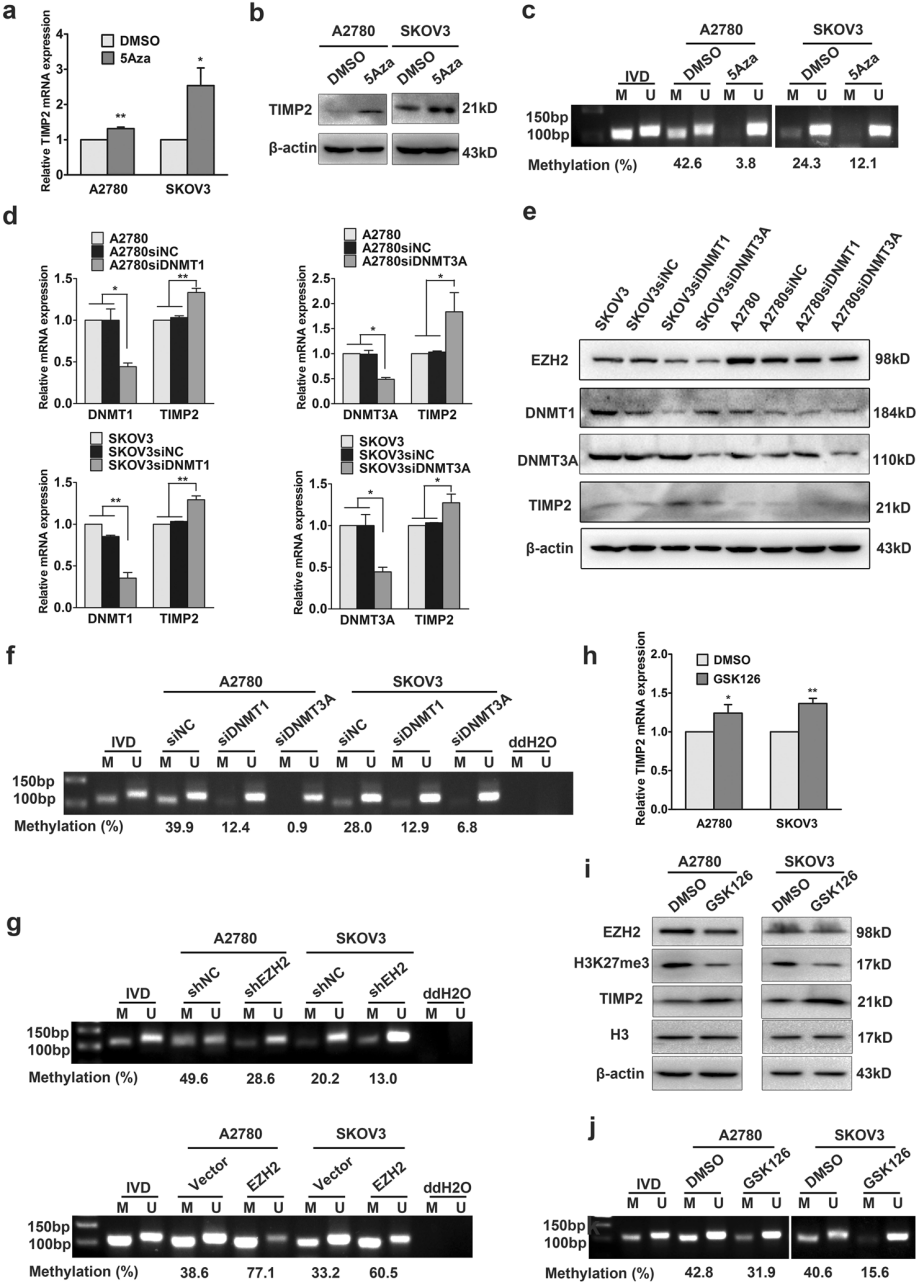
EZH2 inhibits TIMP2 expression via DNA methylation. (**a**) qRT-PCR and (**b**) Western blot analysis of TIMP2 expression in A2780 and SKOV3 cells after treatment with 5Aza. The cropped blots are used in the figure, and full-length blots are presented in Supplementary Fig. S11. (**c**) Methylation-specific PCR (MSP) analysis revealed that 5Aza treatment markedly decreased TIMP2 promoter methylation. The cropped gel is used in the figure, and the full-length gel is presented in Supplementary Fig. S12. M, methylated; U, unmethylated; IVD, *in vitro* methylated DNA; methylation index refers to the percentage of M/M + U. (**d**) qRT-PCR and (**e**) Western blot analysis of TIMP2 expression in A2780 and SKOV3 cells after DNMT1 and DNMT3A siRNA transfection. The cropped blots are used in the figure, and full-length blots are presented in Supplementary Fig. S13. (**f**) MSP for DNA methylation status of the TIMP2 promoter in ovarian cancer cells after DNMT1 and DNMT3A siRNA transfection. The cropped gel is used in the figure, and the full-length gel is presented in Supplementary Fig. S14. (**g**) MSP for DNA methylation status of the TIMP2 promoter in the EZH2-regulated ovarian cancer cells. The cropped gels are used in the figure, and full-length gels are presented in Supplementary Fig. S15. (**h**) qRT-PCR and (**i**) Western blot analysis of TIMP2 expression in A2780 and SKOV3 cells after treatment with GSK126. The cropped blots are used in the figure, and full-length blots are presented in Supplementary Fig. S16. (**j**) MSP analysis of TIMP2 promoter methylation in A2780 and SKOV3 cells after treatment with GSK126. The cropped gels are used in the figure, and full-length gels are presented in Supplementary Fig. S17. *P < 0.05; **P < 0.01; ***P < 0.001.

After confirming that TIMP2 is repressed by EZH2 in ovarian cancer, we subsequently investigated the underlying mechanism. To clarify whether TIMP2 expression is regulated by DNA methylation, the correlation between TIMP2 promoter methylation level and expression level was assessed in ovarian cancer cells. A strong negative correlation was noted between TIMP2 methylation status and mRNA expression (Supplemental Fig. S4). In addition, both 5Aza (Fig. 5a–c) treatment and DNMT1 and DNMT3A knockdown (Fig. 5d–f) increased TIMP2 mRNA and protein expression and reduced DNA methylation of the TIMP2 promoter, suggesting that TIMP2 expression is down-regulated by DNA methylation in ovarian cancer cells. Then, we focused on the effect of EZH2 on TIMP2 promoter methylation. We found that up- and down-regulation of EZH2 led to an increase and decrease in TIMP2 promoter methylation, respectively (Fig. 5g). Furthermore, GSK126, a highly selective inhibitor of EZH2 methyltransferase activity, was used to assess whether EZH2 regulates TIMP2 via a PRC2-dependent mechanism. GSK126 treatment led to increased TIMP2 expression and decreased promoter methylation (Fig. 5h–j). All these results indicated that both DNA methylation and H3K27me3 are involved in EZH2-mediated TIMP2 inhibition.

**Figure 6:**
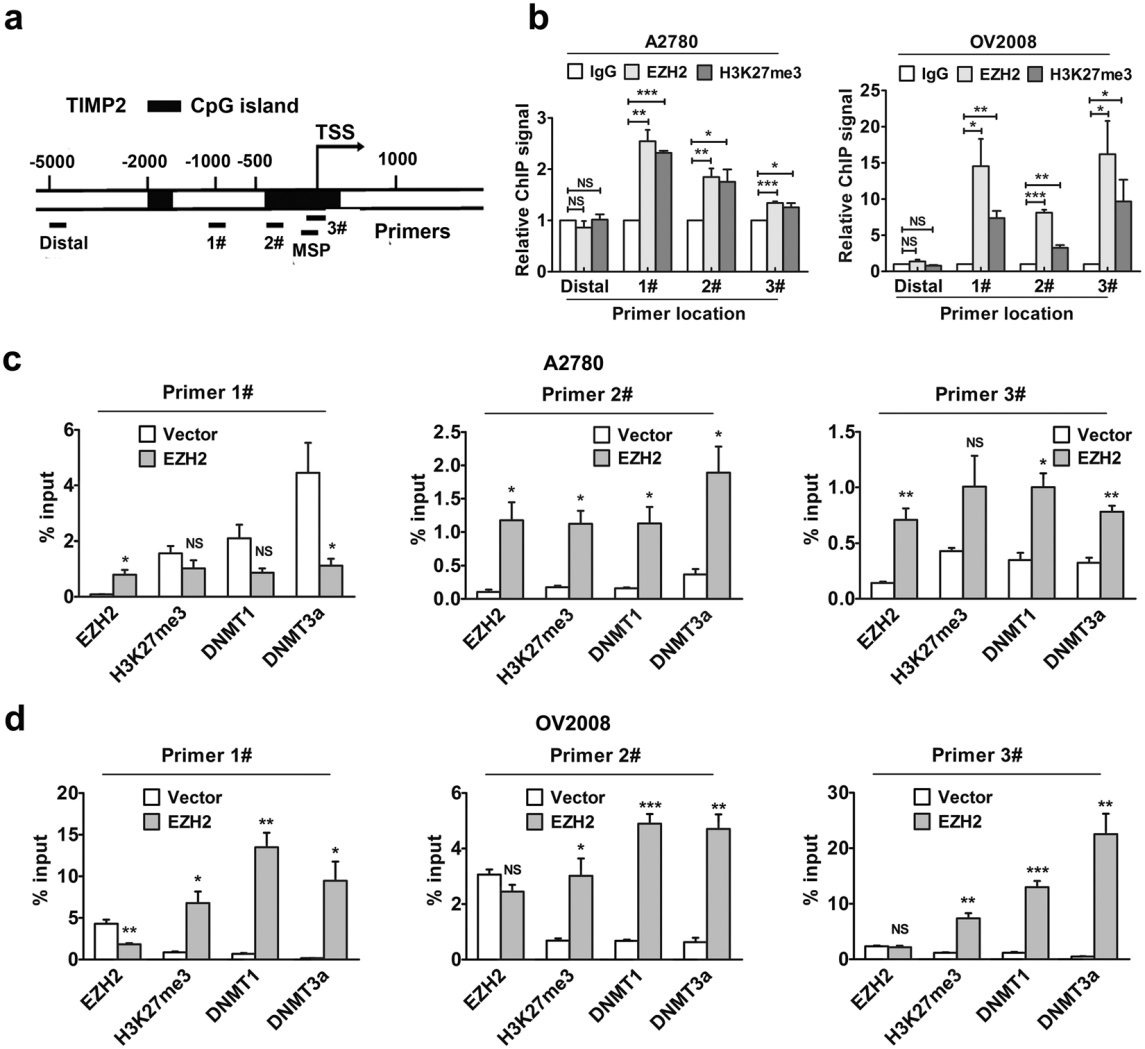
EZH2 recruits DNMTs at the TIMP2 promoter. (**a**) Location of binding sites of primers for MSP and qRT-PCR following ChIP in the context of CpG islands at the TIMP2 promoter. TSS, Transcription start site. (**b**) qRT-PCR analysis of immunoprecipitated chromatin by EZH2 and H3K27me3 antibodies. IgG served as a negative control. (**c**) A2780 and (**d**) OV2008 cells were transfected with EZH2 overexpression plasmid or control followed by ChIP and qRT–PCR for the quantitative detection of EZH2, H3K27me3, DNMT1 and DNMT3a on the TIMP2 promoter. The results were normalized to the input. *P < 0.05; **P < 0.01; ***P < 0.001. NS, not significant.

To further verify the epigenetic silencing of TIMP2 by EZH2, we performed ChIP assays to examine whether EZH2 and H3K27me3 occupy the TIMP2 promoter and the effects of EZH2 on the enrichment of DNMTs at the TIMP2 promoter. The primer binding site locations are illustrated in the map presented in Fig. 6a. The binding sites of primers #1, #2 and #3 are located in the promoter region of TIMP2. Among them, primers #2 and #3 are localized within the CpG island. The distal primer served as a control. The RT–PCR results showed that the TIMP2 promoter was enriched with endogenous EZH2 and H3K27me3 (Fig. 6b). Moreover, dramatic increases in the amount of EZH2, H3K27me3 and DNMT1 and DNMT3a occupying the TIMP2 promoter at the binding sites of primers #2 and #3 were observed when EZH2 was up-regulated in A2780 cells (Fig. 6c). In OV2008, the enrichment of H3K27me3, DNMT1 and DNMT3a at all three primer binding sites in the TIMP2 promoter was significantly increased after EZH2 up-regulation (Fig. 6d). However, EZH2 enrichment at the TIMP2 promoter in OV2008 was not influenced by EZH2 up-regulation, which may be attributed to the high endogenous EZH2 occupancy (Fig. 6b). These findings confirmed the mechanism by which EZH2 acts on the TIMP2 promoter and recruits DNMTs, inducing local abundance of H3K27me3 and DNA methylation and subsequent TIMP2 expression inhibition.

### Inhibition of EZH2 converts histone modification at the TIMP2 promoter

**Figure 7:**
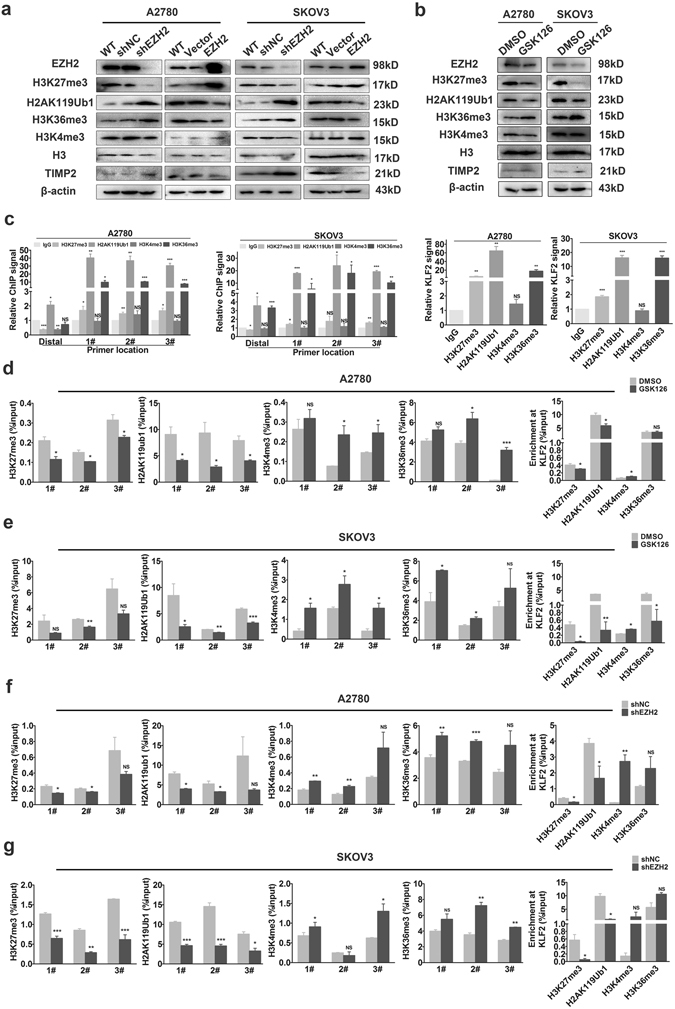
Inhibition of EZH2 converts histone modification mark occupancy at the TIMP2 promoter. (**a**) Western blot analysis of different histone modification marks in A2780 and SKOV3 cells after EZH2 knockdown or pharmacological inhibition by GSK126 treatment (**b**). The cropped blots are used in the figure, and full-length blots are presented in Supplementary Figs S18 and S19. (**c**) ChIP analysis of different histone modification marks at the TIMP2 promoter in A2780 and SKOV3 cells. IgG served as a negative control. (**d**,**e**) ChIP analysis of different histone modification marks at the TIMP2 promoter in A2780 and SKOV3 cells treated by GSK126. (**f**,**g**) ChIP analysis of different histone modification marks in EZH2-knockdown A2780 and SKOV3 cells. The results were normalized to the input. The EZH2 target gene KLF2 was used as a positive control. *P < 0.05; **P < 0.01; ***P < 0.001. NS, not significant.

Given the various histone modifications and the complex crosstalk between different histone modifications and DNA methylation^22^, we detected the change of the “active” histone modifications H3K4me3 and H3K36me3 and the “repressive” H3K27me3 and H2AK119 mono-ubiquitination (H2AK119ub1) at the TIMP2 promoter when EZH2 was down-regulated. Western blotting revealed that EZH2 depletion led to a significant decrease in H3K27me3 and increases in H2AK119ub1, H3K36me3, and TIMP2 and vice versa. No significant impact on H3K4me3 level was observed. (Fig. 7a). Furthermore, GSK126 treatment decreased H3K27me3 and H2AK119ub levels and increased TIMP2 and H3K36me3 levels (Fig. 7b). We evaluated the occupancy of these histone markers at the TIMP2 locus by ChIP assays. The EZH2-target gene KLF2^23^ was used as a positive control. As shown in Fig. 7c, H3K27me3, H2AK119Ub1 and H3K36me3, but not H3K4me3, could occupy the TIMP2 promoter in parent A2780 and SKOV3 cells. Both shEZH2 and GSK126 decreased the repressive H3K27me3 and H2AK119Ub1 but increased the active H3k36me3 and H3K4me3 markers at the TIMP2 promoter (Fig. 7d–g). These results indicated that multiple histone modifications are involved in the EZH2-mediated TIMP2 silencing in ovarian cancer cells.

## Discussion

EZH2 is a key epigenetic modifier and functions as a potential oncogene in a variety of solid tumors. However, the mechanism by which EZH2 promotes tumor invasion remains elusive. Here, we found that high EZH2 expression was associated with metastasis and poor survival in EOC patients and identified TIMP2 as a target of EZH2. EZH2 suppressed TIMP2 expression and increased MMP activity, which subsequently promoted ovarian cancer cell migration and invasion *in vitro* and *in vivo*. Moreover, we clarified that EZH2 inhibits TIMP2 expression by promoting H3K27me3 occupancy and DNA methylation at the TIMP2 promoter. These findings implicate EZH2 in forming a tumor microenvironment that is favorable for ovarian cancer metastasis.

EZH2 expression is elevated in a variety of malignant tumors, including prostate, breast, lung, bladder and hepatocellular cancer^6, 7, 24–26^. We found that both EZH2 mRNA and protein levels were up-regulated in ovarian cancer compared with normal tissue. EZH2 expression in tumors was negatively regulated by several miRNAs, such as miR-101 and let-7e in ovarian cancer^27, 28^. A recent study highlighted the role of NF-YA in regulating EZH2 transcription by binding to two CCAAT boxes in the proximal EZH2 promoter^29^. In lymphoma, a heterozygous missense mutation at amino acid Y641 was identified. This mutation confers enhanced EZH2 catalytic activity for H3K27me3 and is thus functionally equivalent to EZH2 overexpression^30^. A high EZH2 level is often correlated with advanced disease. In ovarian cancer, high EZH2 expression was significantly correlated with tumor angiogenesis, progression and metastasis of ovarian cancer^10, 31, 32^. Consistent with previous studies, we found that EZH2 overexpression was associated with higher FIGO stage and tumor metastasis. Accumulating evidence reveals that EZH2 promotes tumor progression by PRC2-mediated epigenetic silencing of critical tumor suppressor genes, such as AMD1^8^, E-cadherin^33^, and RKIP^24^. Knockdown of EZH2 expression in ovarian cancer cells led to decreased H3K27me3 levels and suppressed cell proliferation and invasion *in vitro* and *in vivo* ^10, 11^. These findings support the oncogenic role of EZH2 in ovarian cancer.

We showed that down-regulation of TIMP2 could reverse the reduced invasion and migration by EZH2 depletion *in vitro* and *in vivo*. Transwell invasion assays were used to evaluate the ability of cells to invade through a Matrigel basement membrane matrix predominantly containing laminin and collagen IV, which are substrates of MMP2 and MMP9. The results of invasion assays were consistent with the function of TIMP2 as a MMP inhibitor. The migration assays detected cell mobility independent of ECM degradation. Indeed, TIMP2, MMP2, and MMP9 are factors with multiple functions and have been implicated in cell invasion, migration, proliferation, apoptosis, angiogenesis, and immune-suppression in tumor^34, 35^. In addition to ECM, MMP2 and MMP9 can cleave cytokines, growth factors, chemokines and receptors of cytokines involved in tumor progression^36^. Moreover, MMP inhibition-independent mechanisms contribute to the multiple biological activities of TIMP2. TIMP2 inhibited vascular endothelial growth factor A (VEGF-A)-induced endothelial cell proliferation and angiogenesis by binding to α3β1 integrin^37^. Moreover, TIMP2 could prevent the activation of tyrosine kinase receptors in tumor cells, including focal adhesion kinase^38^, AKT^39^ and epithelial growth factor receptor^40^, which play key roles in tumor migration and growth. Interestingly, EZH2 has a similar functional spectrum to that of TIMP2 with regard to the regulation of tumor biology and plays an active role in metastasis and angiogenesis in ovarian cancer. This evidence suggests a significant contribution of TIMP2 to EZH2 function in ovarian cancer.

Gene expression is determined, in part, by the accessibility to the transcriptional machinery and factors. Epigenetic modifications, such as DNA methylation, DNA deacetylation, and histone methylation, repress gene expression by changing the local chromatin configuration at gene promoters and enhancers that impair the DNA accessibility^41^. Together with SUZ2 and EED, EZH2 forms the PRC2 that catalyzes the H3K27me3 repressive chromatin mark^4^. H3K27me3 at promoter regions recruits DNMTs to CpG islands, leading to DNA methylation that further decreases accessibility and represses gene expression^5^. We found that the inhibition of H3K27me3 by GSK126 treatment reduced DNA methylation at the TIMP2 promoter. Moreover, ChIP assays revealed that EZH2 overexpression resulted in significant increases in H3K27me3 and DNMTs at the TIMP2 promoter. Both GSK126 and 5Aza increased TIMP2 transcription. These findings suggest that both H3K27me3 and DNA methylation contribute to EZH2-mediated TIMP2 silencing. DNA hypermethylation-mediated silencing of TIMP2 was described in different types of tumors, including neuroblastomas, prostate cancer, glioblastoma and breast carcinoma^18, 42, 43^. However, the role of histone methylation in TIMP2 expression regulation was reported only in prostate cancer cells. Specifically, EZH2 represses the expression of TIMP2 and TIMP3 via H3K27me3 and subsequent DNA methylation^19^. Notably, in HO8910 cells, the TIMP2 promoter was highly methylated, whereas EZH2 expression and H3K27me3 were low, indicating that the high methylation status of TIMP2 in HO8910 may involve mechanisms other than pre-existing H3K27me3, which requires further research.

Polycomb group proteins (PcG) consist of two major complexes, polycomb repressive complex 1 and 2 (PRC1 and PRC2), which play a critical role in repressing gene expression. PRC1 modifies histone H2A at Lys119 by adding a ubiquityl moiety, whereas PRC2 methylates histone H3 at Lys27 by catalyzing the addition of one to three methyl groups^44, 45^. PRC1 and PRC2 interact to repress gene expression in a synergistic manner^45^. In contrast, methylation on H3 at Lys4 (H3K4) and Lys 36 (H3K36) is antagonistic to H3K27me3 and is generally associated with gene activation^45^. Nucleosomes pre-methylated at H3K27 are largely refractory to enzymatic catalysis at H3K36 and vice versa^46, 47^. We found that both down-regulation of EZH2 and pharmacological inhibition of H3K27me3 by GSK126 treatment led to re-activation of TIMP2 expression, accompanied by decreased H3K27me3 and H2AK119Ub1 and increased H3K36me3 and H3K4me3 occupancy at the TIMP2 promoter (Fig. 7d,e). These findings suggest that EZH2 represses TIMP2 transcription via multiple histone modifications that can led to chromatin condensation and DNA methylation. In addition, our results suggest that in cells where EZH2 is absent, H3K36me3 and H3K4me3 may contribute to the transcriptional activation of TIMP2. Moreover, the H2AK119ub1 level was increased by EZH2 depletion and decreased by EZH2 overexpression, while GSK126 treatment partially inhibited H2AK119ub1 (Fig. 7a,b). PRC1 and PRC2 are known reinforce each other, as the enzymatic activity of each complex enhances the occupancy and activity of the other on chromatin^45^. This cooperative interplay between PRC1 and PRC2 was mirrored by the decrease in H2AK119ub1 induced by GSK126 (Fig. 7a). The paradox of H2AK119ub1 and H3K27me3 alterations mediated by EZH2 depletion or overexpression may be due to the PRC2-independent activities of EZH2, which require further investigation.

In conclusion, EZH2 promotes ovarian cancer migration and invasion via the epigenetic silencing of TIMP2 by H3K27me3 and DNA hypermethylation, which leads to the activation of MMP2 and MMP9. These findings link EZH2 and H3K27me3 to tumor ECM degradation and provide new insights into the mechanisms underlying the oncogenic function of EZH2 in ovarian cancer.

## Materials and Methods

### Ethical Statement

This study was conducted according to the principles expressed in the Declaration of Helsinki and was approved by the Research Ethics Committee of Union Hospital, Tongji Medical College, Huazhong University of Science and Technology (Wuhan, China). Written informed consent was obtained from all patients. The procedures were carried out in accordance with the university’s scientific research guidelines and regulations.

### Bioinformatics analysis

Level 3 HiSeq RNASeq data including 568 primary ovarian serous cystadenocarcinoma samples and 8 normal tissues was downloaded from The Cancer Genome Atlas website (TCGA, https://tcga-data.nci.nih.gov/tcga/), and eight different Affymetrix microarray datasets (GSE26712, GSE18520, GSE12470, GSE9891, GSE27651, GSE26193, GSE30161 and GSE49997) were downloaded from the Gene Expression Omnibus website (GEO, http://www.ncbi.nlm.nih.gov/geo/) in view of the relatively large number of samples and complete clinical information. Expressions of EZH2 between normal ovary and ovarian cancer were compared and the relation between EZH2 and clinicopathologic features were studied. KM-Plotter (http://kmplot.com/analysis/index.php?p=service&cancer=ovar), an open free on-line analysis website, was used to illustrate prognosis significance of EZH2 in ovarian cancer patients. “EZH2” was entered as gene symbol, and the “Auto select best cutoff” button was selected to determine cut-off point dividing EZH2 expression as high or low level. Kaplan-Meier and Log-Rank test was used to evaluate the difference in overall survival time. The correlations of EZH2 and TIMP family genes were examined by Pearson’s correlation analysis.

### Ovarian cancer samples

EOC tissues were from paraffin-embedded specimen repository of Department of Gynecology and Obstetrics, Union Hospital, Tongji Medical College, Huazhong University of Science and Technology. In total, 82 patients who received resection surgery at the department between August 2008 and October 2015 were enrolled in this study. No patients received any chemotherapeutic treatment or radiotherapy before surgery, and all of the patients were followed up every three months. Pathological diagnosis was confirmed by at least two pathologists independently. The detailed clinicopathological features of these patients were shown in Table 1. The use of ovarian cancer tissues was approved by the Research Ethics Committee of Union Hospital, Tongji Medical College, Huazhong University of Science and Technology (Wuhan, China).

### Tissue microarray and immunohistochemistry (IHC)

The formalin-fixed, paraffin-embedded human ovarian cancer tissue array containing 82 duplicated cancer tissues was used for immunohistochemistry (IHC) examination of EZH2 and TIMP2. IHC was performed as previously described^11^. Slides were dehydrated through a graded series of alcohols followed by antigen retrieval in 0.01 M citrate buffer at 95 °C for 20 min. They were then incubated in 3% H_2_O_2_ for 20 min to inactivate endogenous peroxidase and blocked in 5% BSA for 20 min at room temperature. Then, the slides were incubated with primary antibody against EZH2 (1:200 dilution, Cell Signaling Technology, Danvers, MA, USA) and TIMP2 (1:200 dilution, R&D Systems, Minneapolis, MN, USA) overnight at 4 °C. The next day, the slides were washed with phosphate buffer solution (PBS) and incubated with biotinylated secondary antibody for 20 min at 37 °C. Fresh 3,3-diaminobenzidin (DAB) solution was used to visualize the target proteins. Two observers independently evaluated the expressions of target proteins. EZH2 immunoreactivity was assessed according to the average percentage of positively stained cells in five randomly selected fields. The immunoreactivity score of TIMP2 was determined by multiplying the intensity score by the proportion score. Intensity was scored semi-quantitatively as negative (score 0), weak (score 1), moderate (score 2) or strong (score 3), and the proportion of positive cells was scored as negative (score 0), <10% (score 1), 11–50% (score 2), 51–80% (score 3) and >80% (score 4). Greater than 25% cells positive for EZH2 and a TIMP2 immunoreactivity score greater than 4 was considered high expression.

### Cell culture and chemical reagent treatment

EOC cell lines A2780, CAOV3, C13*, ES2, HO8910, OV2008 and SKOV3 were purchased from China Center for Type Culture Collection (CCTCC, Wuhan, China). All cells were cultured at 37 °C with DMEM/F12 medium (Gibco, Grand Island, NY, USA) containing 10% fetal calf serum (Gibco, USA) under 5% CO2 atmosphere. The demethylation agent 5-Azacytidine (5Aza, Sigma-Aldrich Corp, St. Louis, MO, USA) and the inhibitor of H3K27me3-GSK126 (Cayman Chemical, Ann Arbor, Michigan, USA) were added to fresh culture medium of ovarian cancer cells with final concentrations of 10 uM and 500 nM, respectively. Cells were collected and analyzed by subsequent experiments after 72 h of culture.

### Transfection *in vitro*

Lentiviruses containing EZH2 shRNA, non-targeting scrambled shRNA (shNC), TIMP2 siRNA, DNMT1 siRNA, DNMT3A siRNA and a scramble non-targeting siRNA (siNC) were purchased from GenePharma (Suzhou, China). The EZH2-shRNA sequence was as follows: 5′-GCAACACCCAACACTTATAAG-3′. The TIMP2 siRNA sequence was as follows: 5′-GGAAAGAAGGAAUAUCUCAdTdT-3′. The DNMT1 siRNA sequence was as follows: 5′-GGAAGAAGAGUUACUAUAA-3′. The DNMT3A siRNA sequence was as follows: 5′- GCACUGAAAUGGAAAGGGUUU-3′. The EZH2-overexpressing and TIMP2-overexpressing plasmid (GV166-EZH2 plasmid, pCMV-N-Flag-TIMP2 plasmid) were constructed in our laboratory. Full-length EZH2 and TIMP2 cDNAs were cloned into GV166 and pCMV-N-Flag plasmids. Stable transfection of EZH2 knockdown lentivirus was conducted following the instructions. Transfected cells were selected with puromycin followed by an isolation of monoclone for further culture. Transient transfection of siRNA and plasmids were conducted using Lipofectamine 2000 transfection reagent (Invitrogen, Carlsbad, CA, USA) according to the manufacturer’s instructions.

### Quantitative real-time PCR (RT-PCR)

RNA extraction and quantitative real-time PCR were conducted as described previously^48^. Briefly, total cellular RNA was extracted with TRIzol (Invitrogen, Carlsbad, CA, USA) according to the manufacturer’s instruction. RNA quality and quantity were detected with a NanoDrop 2000/2000C instrument (Thermo Scientific, CA, USA). cDNA was synthesized using a Reverse Transcription Kit (Toyobo, Osaka, Japan). PCR reactions including standard curve analysis were performed on an Applied Biosystem StepOne Plus PCR system (ABI, USA) using SYBR Green Real-time PCR Master Mix (TaKaRa, Otsu, Japan). All primers were designed based on NCBI reference sequences and synthesized by Invitrogen (Suzhou, China). Primer sequences were listed in Supplemental Table S1. Each pair of primers was first assessed by a standard curve to ensure the amplification efficiencies were approximately 100%. The two-step PCR program was as follows: 95 °C for 1 min, 40 cycles of 95 °C for 20 s, 60 °C for 20 s, and 70 °C for 10 s. Target gene expression was normalized to β-actin and calculated using the 2^−ΔΔCt^ method. Each experiment was performed in triplicate at least three times.

### Western blot analysis

Total cell protein was extracted with radioimmunoprecipitation assay (RIPA) buffer (Beyotime Biotechnology, Shanghai, China) including a protease inhibitor cocktail for 30 min and centrifuged at 13,000 g for 10 min to collect supernatants. The protein concentration was measured with a bicinchoninic acid (BCA) assay, and then, samples were separated by 10% sodium dodecyl sulfate-PAGE (SDS-PAGE) and transferred onto a PVDF membrane. The membranes were then blocked with 5% nonfat milk in Tris-buffered saline with Tween-20 (TBST) for 1 h at room temperature and incubated with primary antibody against EZH2 (1:2000 dilution, CST, USA), TIMP2 (1:1000 dilution, CST, USA), Histone 3 (1:3000 dilution, CST, USA), H3K27me3 (1:1000 dilution, ABclonal, Woburn, MA, USA), MMP2 (1:100 dilution, Santa Cruz Biotechnology, CA, USA), MMP9 (1:1000 dilution, Abcam, Cambridge, UK), DNMT1 (1:500 dilution, Santa Cruz Biotechnology, CA, USA), DNMT3A (1:500 dilution, Santa Cruz Biotechnology, CA, USA), Ubiquitin-Histone H2A (Lys119) (1:2000 dilution, CST, USA), Histone H3K4me3 (1:1000 dilution, Affinity Biosciences, OH, USA), Histone H3K36me3 (1:1000 dilution, Affinity Biosciences, USA) and β-actin (1:5000 dilution, CST, USA) in 4 °C overnight. Horseradish peroxidase-conjugated anti-rabbit, anti-mouse or anti-goat secondary antibodies (1:5000 dilution, CST, USA) were subsequently used. Protein bands were detected with an enhanced chemiluminescence kit (Pierce, Thermo Scientific, Waltham, MA, USA) in Molecular Imager® ChemiDocTM XRS+ with Image Lab^TM^ Software (Bio-Rad Laboratories, Hercules, CA, USA).

### Immunocytochemistry

Immunocytochemistry of EZH2 and TIMP2 in ovarian cancer cells was conducted as previously described^11^. The cells were fixed with 4% paraformaldehyde for 30 min at room temperature. Then, 0.1% Triton-X 100 and 0.3% H_2_O_2_ were used to break the cell membrane and inhibit endogenous peroxidase activity, respectively. After blocking with 5% BSA at room temperature for 30 min, cells were incubated with primary anti-EZH2 (1:200 dilution, CST, USA) or anti-TIMP2 (1:200 dilution, R&D, USA) antibodies at 4 °C overnight, followed by incubation with biotinylated goat anti-rabbit (for EZH2) or rabbit anti-goat secondary antibody (for TIMP2) (1:100 dilution) for 1 h at room temperature. Normal rabbit IgG (Santa Cruz, USA) was used as a negative control. Immunostaining was performed with diaminobenzidine (DAB). Positive expression was defined as brown-yellow granules distributed in the cytoplasm or nucleus.

### Gelatin zymography assay

Confluent ovarian cancer cells were maintained in serum-free media for 24 h and the culture media was collected and analyzed using the gelatin zymography assay. Equal amounts of media were mixed with the SDS sample buffer without β-mercaptoethanol and loaded onto 10% SDS-PAGE gels containing 0.1% porcine gelatin (Sigma, USA). Electrophoresis was performed in an ice bath to prevent enzyme activation. Gels were then washed 4 times with gentle agitation for 15 min each with elution buffer (2.5% Triton X–100, 50 mmol/L Tris-HCL, 5 mmol/L CaCl2 and 1 μmol/L ZnCl2, pH 7.6) and 2 times for 20 min each with wash buffer (50 mmol/L Tris-HCL, 5 mmol/L CaCl2 and 1 μmol/L ZnCl2, pH 7.6). Gels were then placed in incubation buffer (50 mmol/L Tris-HCL, 5 mmol/CaCl2, 1 μmol/L ZnCl2 and 0.05% NaN3) and incubated at 37 °C for 48 h. Finally, gels were stained with staining buffer (0.05% Coomassie brilliant blue R-250, 30% methanol and 10% acetic acid) for 3 h and destained with destaining buffer (30% methanol and 10% acetic acid) for 3 h at room temperature. The bands were detected and quantified with Image Lab^TM^ Software.

### Cell invasion and migration assay

Cells were trypsinized, counted and resuspended in serum-free DMEM/F12, and 40,000 and 80,000 cells were used for the migration and invasion assays, respectively. Briefly, 200-µl cell suspensions were plated in the upper non-Matrigel-coated chamber or Matrigel-coated chamber (8-mm pore size, Millipore, Billerica, MA, USA). Fresh media containing 10% FBS was added to the lower chamber. After a 24-h incubation, the cells in the upper chamber were wiped with cotton swabs. The cells on the filter surface were fixed with methanol, stained with crystal violet, and imaged under microscopy. The average numbers of migrated or invaded cells in three random fields per well were obtained.

### Scratch-wound assay

Cells were seeded in 6-well plates to approximately 100% confluence. A linear scratch wound was created in the confluent monolayer using a 200-μl pipette tip. After washing the cells gently with phosphate buffer solution (PBS) to remove suspended cells, the culture medium was replaced with serum-free medium to block cell proliferation, and cancer cells were allowed to close the wound for 24 h. The closure area was determined by comparing the images from the start time point (0 h) to the last time point (24 h) under microscopy using Image-Pro Plus 6 software.

### Intraperitoneal xenografts model

All *in vivo* experiments were performed according to the guidelines and protocols for animal care approved by the Tongji Medical College’s Animal Care and Use Committee. Female BALB/c nude mice (Beijing Vital River, China) at four to six weeks of age were used for the mouse model. Cholesterol-conjugated TIMP2 siRNA for *in vivo* siRNA delivery was modified and purchased from GenePharma (Suzhou, China). The ovarian cancer xenografts were derived from SKOV3 cells that were stably transfected with shNC or shEZH2. Three days before tumor cell injection, half of the SKOV3-shEZH2 cells were transiently transfected with TIMP2 siRNA (co-transfection group). Every 3 × 10^6^ SKOV3-shNC cells, SKOV3-shEZH2 cells or co-transfected cells were resuspended in 300 μl PBS. Eighteen nude mice were divided randomly into three groups and intraperitoneally injected (i.p.) with cancer cells. Four days after the inoculation, the mice in co-transfection group received an *in vivo* transfection of siTIMP2. Then 50 µg/g cholesterol-conjugated siRNA was diluted in saline buffer and injected intraperitoneally once per week. Another two groups were injected with saline buffer (once per week, 300 µl/mouse). All mice were sacrificed 4 weeks after tumor inoculation. All tumors were recorded and dissected immediately after death and weighed. The xenograft tissues were divided into two sections. One part was embedded with paraffin for hematoxylin–eosin staining and histopathological analysis for EZH2 and TIMP2, and another was conserved in liquid nitrogen cryopreservation for extraction of tissue mRNA and protein.

### Bisulfite modification and methylation-specific PCR (MSP)

To predict CpG islands in the TIMP2 promoter, we searched the TIMP2 genomic sequence including two kb upstream and one kb downstream of the transcriptional start site (TSS) using the NCBI website (http://www.ncbi.nlm.nih.gov/nuccore). The sequences were assessed with DataBase of CpG islands and Analytical Tool (DBCAT, http://dbcat.cgm.ntu.edu.tw/) according to the following criteria: the length of the CpG island >200 bp, observed/expected CpG ratio >0.6 and percentage of G plus C >50%. According to the predicted CpG islands and published literature, we developed one pair of nested MSP primers^49^ that is listed in Supplemental Table S1. Genomic DNA of ovarian cancer cells was extracted using a DNA Isolation Kit (Tiangen Biotech, Beijing, China). Bisulfite modification of DNA was performed following the recommended procedures of the BisulFlash DNA Modification Kit (Epigentek, Farmingdale, NY,USA). Placenta DNA was modified with the CpG Methyltransferase (M.SssI, NewEngland Biolabs, Ipswitch, MA, USA) and used as a positive control. Every 300 ng of genomic DNA was bisulfite-treated and then quantified with a NanoDrop 2000/2000C system (Thermo Scientific, USA). Modified DNA was first amplified with flanking primers followed by specific methylated or unmethylated primers using PrimeScript™ RT Master Mix (TaKaRa, Otsu, Japan). MSP products were subjected to electrophoresis on 3% agarose gels that were stained with ethidium bromide, detected and analyzed with Image Lab^TM^ Software.

### Chromatin immunoprecipitation assay (ChIP)

The ChIP assay was performed according to the manufacturer’s instructions for the EpiQuik Chromatin Immunoprecipitation (ChIP) Kit (Epigentek, Farmingdale, NY, USA). Primary antibodies against EZH2 (CST, USA), H3K27me3 (ABclonal, USA), H2AK119Ub1 (CST, USA), H3K4me3 (Affinity Biosciences, USA), H3K36me3 (Affinity Biosciences, USA), DNMT1 (Santa Cruz Biotechnology, USA) and DNMT3a (Santa Cruz Biotechnology, USA) were used for co-immunoprecipitation. RT-PCR was then conducted. Primers for the ChIP assay were determined according to a previous study^18, 23^ and are listed in Supplemental Table S1. Internal normal mouse IgG and anti-RNA Polymerase II in the kit served as positive and negative control, respectively.

### Statistical analysis

Statistical analysis was performed using SPSS 17.0 statistics software and GraphPad Prism 5.0 software. Numerical data was expressed as the mean ± standard deviation. Student’s t-test or one-way analysis of variance was used to describe the differences between two or multiple groups, respectively. Kaplan-Meier survival analysis was used to present the survival differences between divided groups. Pearson’s correlation coefficient was used to identify the correlation between EZH2 and TIMP2 in EOC tissues. *P* < 0.05 was considered significant.

## Electronic supplementary material

Supplementary figures and tables and full length Figures
