# Supplementary material for: EZH2-mediated epigenetic silencing of TIMP2 promotes ovarian cancer migration and invasion
Source: Sci Rep. 2017 Jun 15;7:3568. doi: 10.1038/s41598-017-03362-z (PMC5472630; doi:10.1038/s41598-017-03362-z)
Supplement: Supplementary file 1 — Supplementary figures and tables and full length Figures [file 41598_2017_3362_MOESM1_ESM.pdf]

EZH2-mediated epigenetic silencing of TIMP2 promotes ovarian cancer migration and invasion

Xiaoqing Yi[1#], Jianfeng Guo[1#], Jing Guo[1], Si Sun[1], Ping Yang[2], Junjie Wang[3], Yuan Li[1], Lisha Xie[1], Jing Cai[1*] and Zehua Wang[1*]

[1] Department of Obstetrics and Gynecology, Union Hospital, Tongji Medical College, Huazhong University of Science and Technology, Wuhan 430022, China.

[2] Department of Obstetrics and Gynecology, First Affiliated Hospital, School of Medicine, Shihezi University, Shihezi 832008, China.

[3] Department of Obstetrics and Gynecology, Renhe Hospital, Three Gorges University, Yichang 443001, China.

[#] These authors contributed equally to this work.

[*]Correspondence: Zehua Wang or Jing Cai, Department of Gynecology and Obstetrics, Union Hospital, Tongji Medical College, Huazhong University of Science and Technology, 1277 Jiefang Avenue, Wuhan, Hubei Province, 430022, P.R. China. E-mail: zehuawang@163.net (Zehua Wang), caijingmmm@hotmail.com (Jing Cai). Tel/Fax: +86 27 85351649.

Supplementary Figures and Table

Supplemental Figure S1

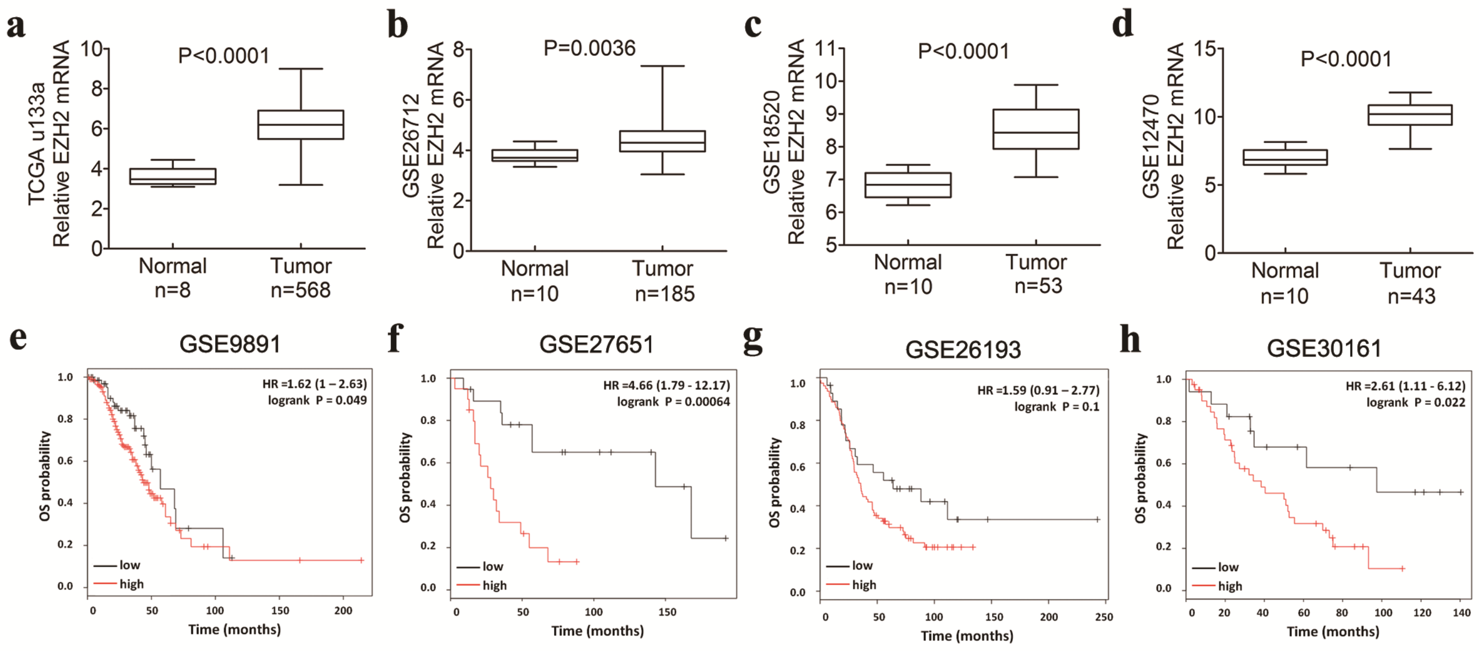

Supplemental Figure S1. High EZH2 expression in ovarian cancer is associated with poor survival in ovarian cancer patients. (a-d) EZH2 mRNA is up-regulated in ovarian cancer tissues compared with normal ovary epithelium in TCGA and GEO databases (GSE26712, GSE18520, and GSE12470). (e-h) Kaplan-Meier analyses of EZH2 expression and overall survival using the online analysis tool-Kaplan-Meier plotter of different GEO data (GSE9891, GSE27651, GSE26193 and GSE30161).

Supplemental Figure S2

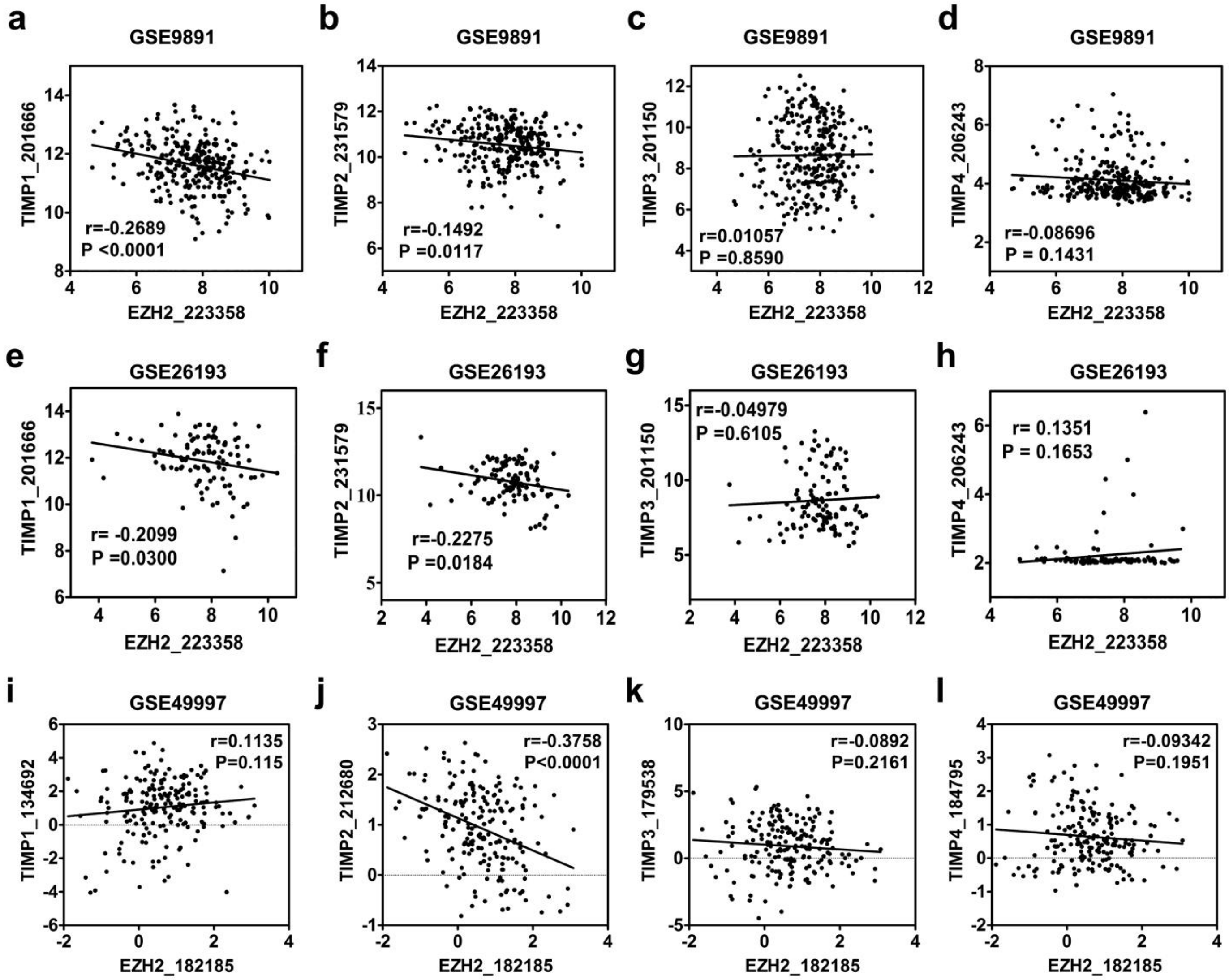

Supplemental Figure S2. EZH2 inversely correlates with TIMP2 expression level in ovarian cancer. (a-d) The correlation between EZH2 and TIMP1, TIMP2, TIMP3 and TIMP4 mRNA levels in GSE9891. (e-h) The correlation between EZH2 and TIMP1, TIMP2, TIMP3 and TIMP4 mRNA levels in GSE26191. (i-l) The correlation between EZH2 and TIMP1, TIMP2, TIMP3 and TIMP4 mRNA levels in GSE49997.

Supplemental Figure S3

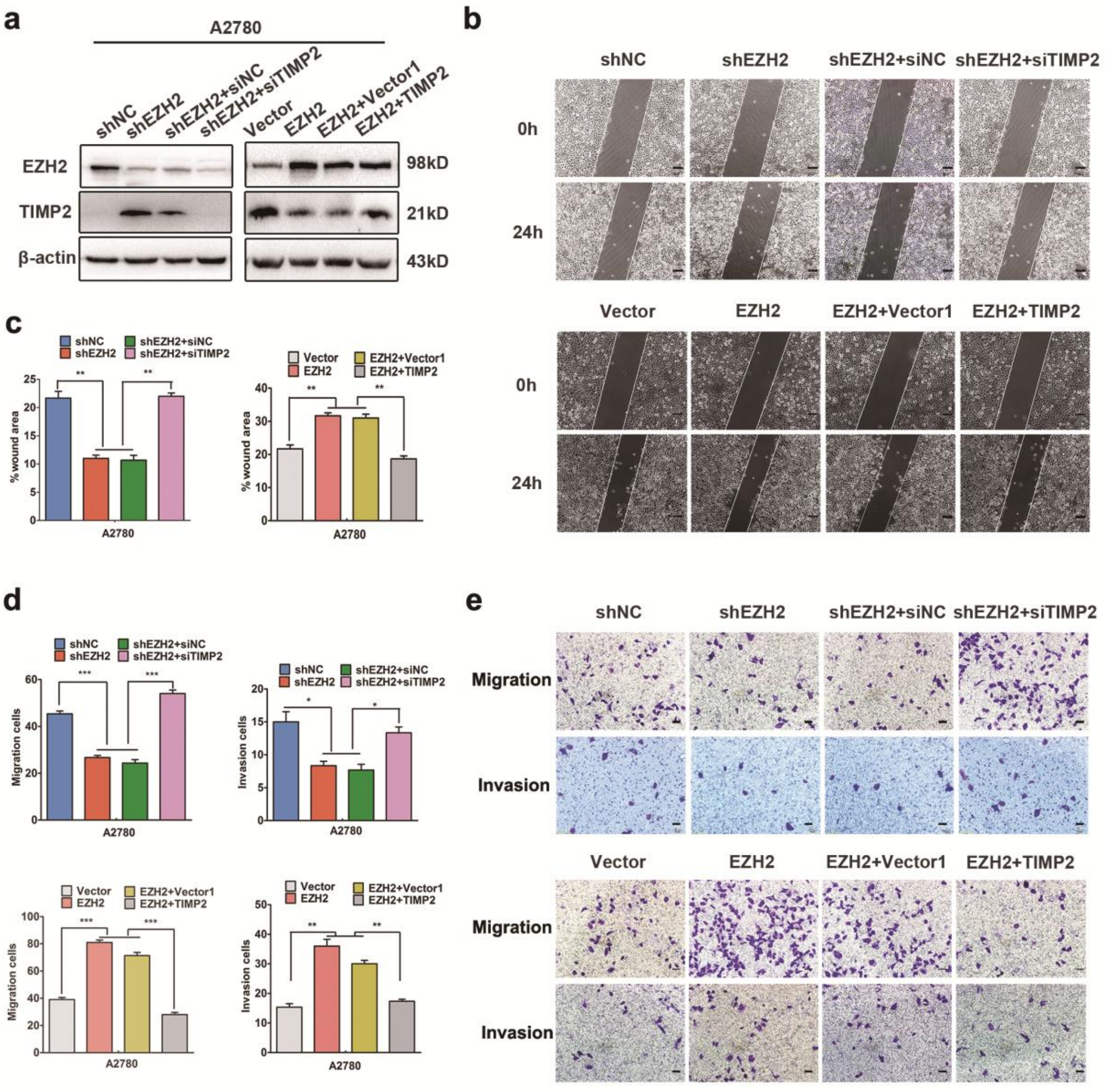

Supplemental Figure S3. EZH2 promotes invasion and migration of A2780 cells by repressing TIMP2. A2780 cells were transfected with shEZH2, shEZH2 and siTIMP2; EZH2-plasmid, EZH2-plasmid and TIMP2-plasmid; and corresponding controls. (a) Western blot analysis of EZH2 and TIMP2 in A2780 cells after co-transfection. (b) Scratch-wound assays for detection of cell mobility (scale bar, 100 μm). (c) Histograms show the wound-healing areas of scratch-wound assays. (d) Transwell migration and invasion assays. The number of cells

exhibiting invasion and migration are presented. (e) Representative images of migration and invasion assays (scale bar, 50 μm). *, $P<0.05$; **, $P<0.01$; ***, $P<0.001$.

Supplemental Figure S4

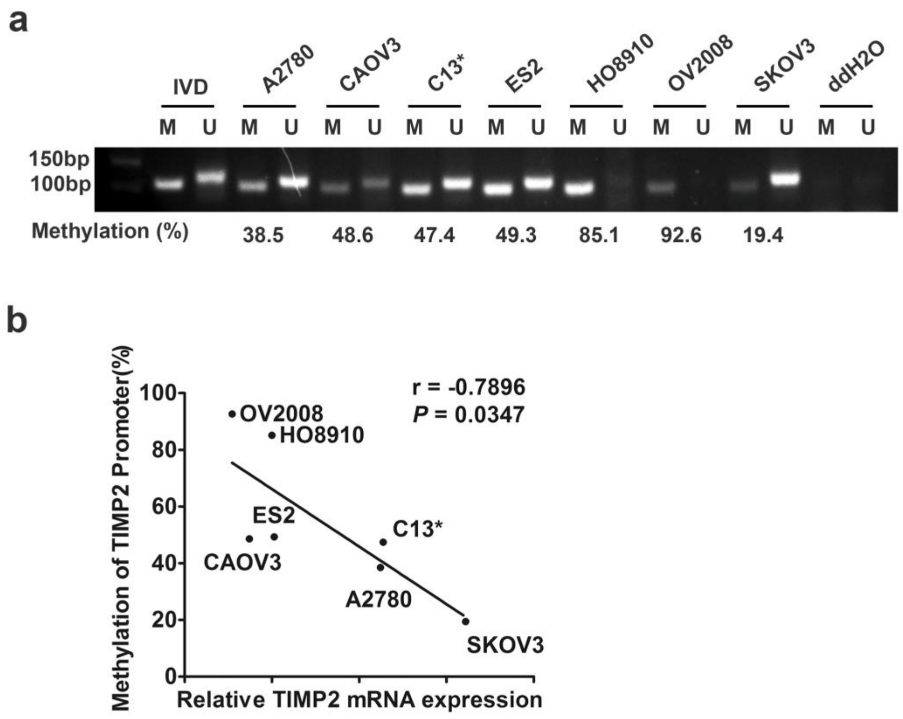

Supplemental Figure S4. TIMP2 expression negatively correlates with DNA methylation in ovarian cancer cell lines. A remarkable negative correlation was noted between TIMP2 methylation status and mRNA expression. (a) Methylation-specific PCR (MSP) for DNA methylation status of the TIMP2 promoter in ovarian cancer cell lines. The cropped gel is used in the figure and full-length gel is presented in **Supplementary Figure S10**. M, methylated; U, unmethylated; IVD, *in vitro* methylated DNA; methylation index refers to the percentage of M/M+U. (b) TIMP2 mRNA level was significantly negatively related to methylation level (Pearson correlation r=-0.7896, $P=0.0347$).

Supplemental Table S1. Primers for Real time PCR , nested MSP ,and q-PCR of CHIP assay

	Forward primer (5'-3')	Reverse primer (5'-3')
**Real time PCR**		
EZH2	TTGTTGGCGGAAGCGTGTAAAATC	TCCCTAGTCCCGCGCAATGAGC
TIMP2	ATAAAGATGTTCAAAGGGCCTG	CCTTCTTTCCTCCAACGTC
β-actin	GCCAACACAGTGCTGTCTGG	GCTCAGGAGGAGCAATGATCTTG
DNMT1	CCATCAGGCATTCTACCA	CGTTCTCCTTGTCTTCTCT
DNMT3A	TATTGATGAGCGCACAAGAGAGC	GGGTGTTCCAGGGTAACATTGAG
**Nested MSP**		
External	TYGGGAGGAGGAGTAGAAAATTT	AACRAAACRACRAAATAAAAAAC
Methylated	TAATAAAATTGCGGTTCGGTTTAAGTTC	CGAAACGACGAAATAAAAAACGACG
Unmethylated	GAGTGTAATAAAATTGTGGTTTGGTTTAAGTTT	CCAAACAAAACAACAAAATAAAAAACAACA
**ChIP**		
Distal	GGATCTGGCCCAAATGTCA	GGCTCATCGTGTTCTATGGT
1#	CCTCTCCCTAGCTGGACTGCAA	GTGGGCACCCACCCTGT
2#	TAGGCCACCCGCCCACAGAAA	GGGTCCCTTCGAGCCCAG
3#	CATTGGCCGCCAGCCAC	GCGCTGCCTTCTACGGATGT
KLF2	GAGACTCCAGACTTCCCATCC	CAGAGACTCTCAGGGGAGCAC

Supplemental Figure S5

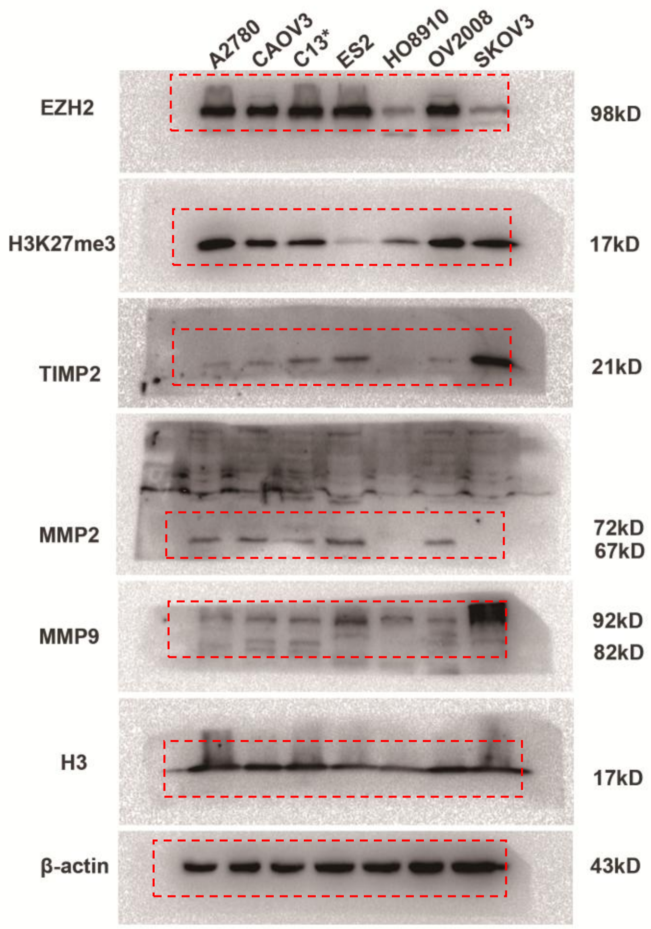

Supplementary Figure S5. Full length blots of Figure 2a. Red dotted lines show the cropping locations.

Supplemental Figure S6

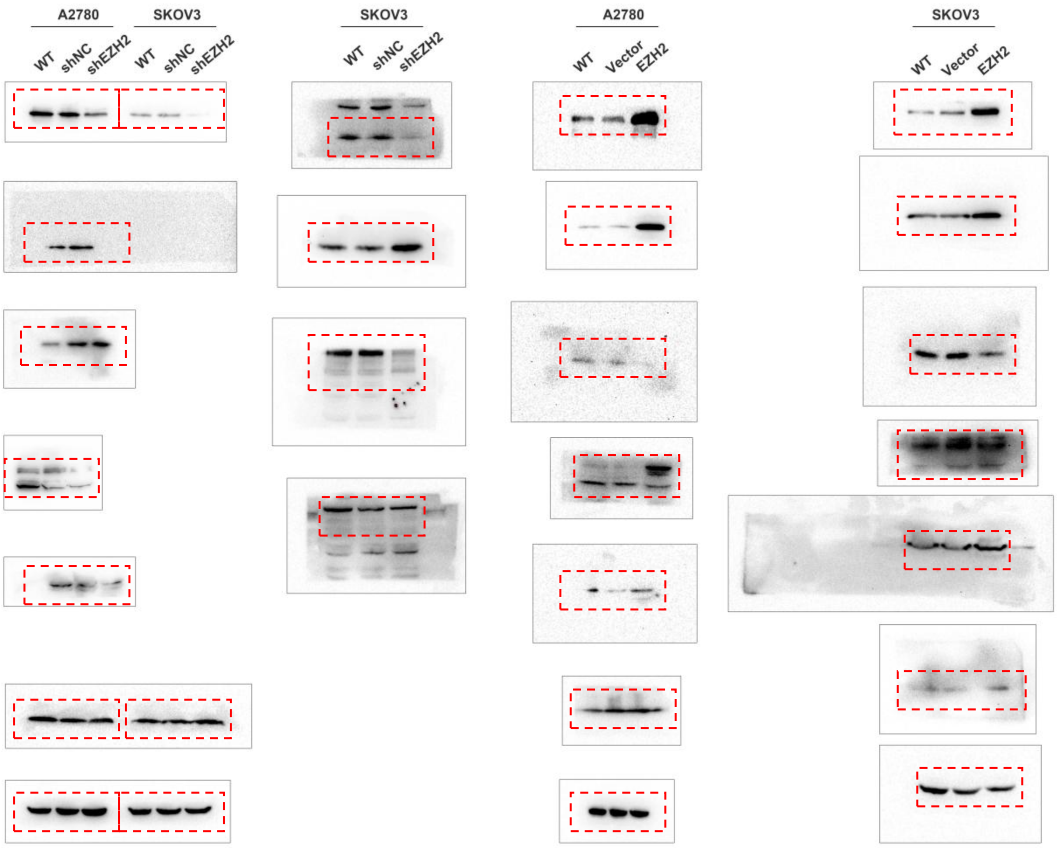

Supplementary Figure S6. Full length blots of Figure 2e. Red dotted lines show the cropping locations.

Supplemental Figure S7

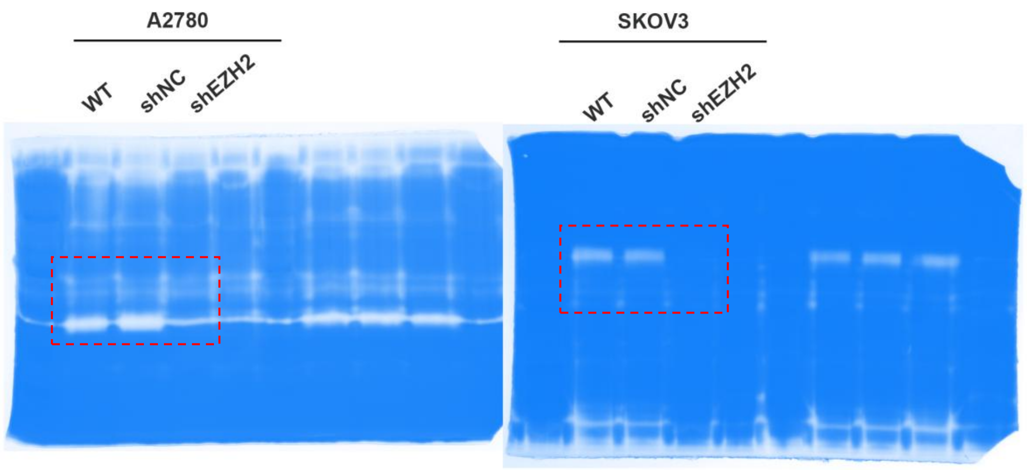

Supplementary Figure S7. Full length gels of Figure 2g. Red dotted lines show the cropping locations. Brightness was adjusted during processing these gels.

Supplemental Figure S8

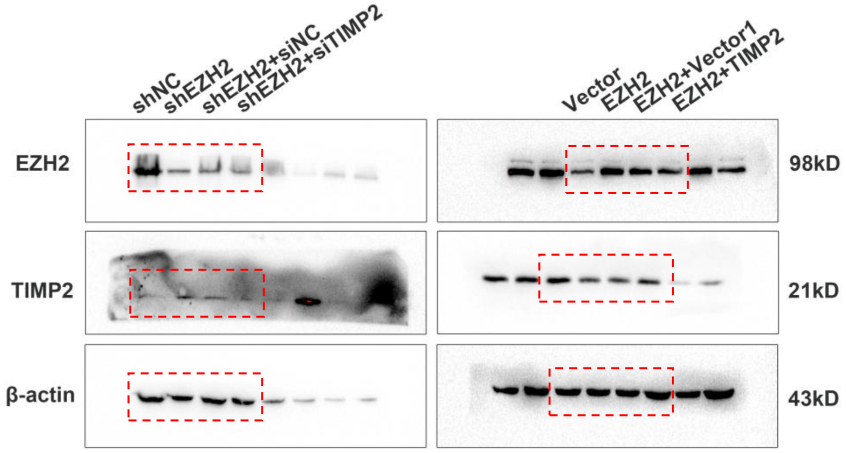

Supplementary Figure S8. Full length blots of Figure 3a. Red dotted lines show the cropping locations.

Supplemental Figure S9

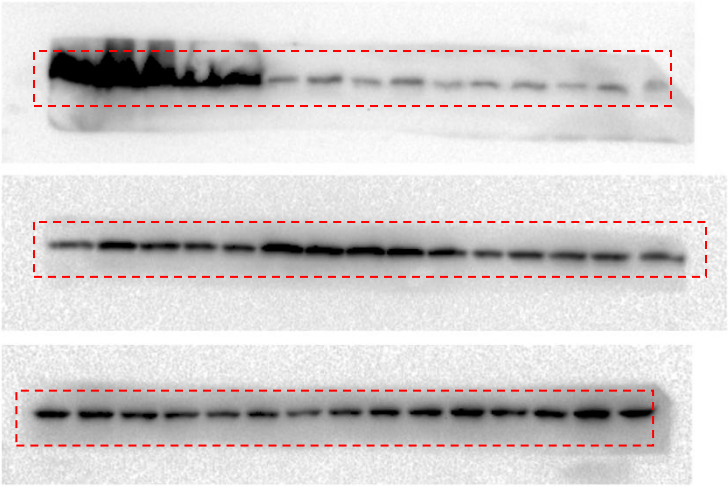

Supplementary Figure S9. Full length blots of Figure 4e. Red dotted lines show the cropping locations.

Supplemental Figure S10

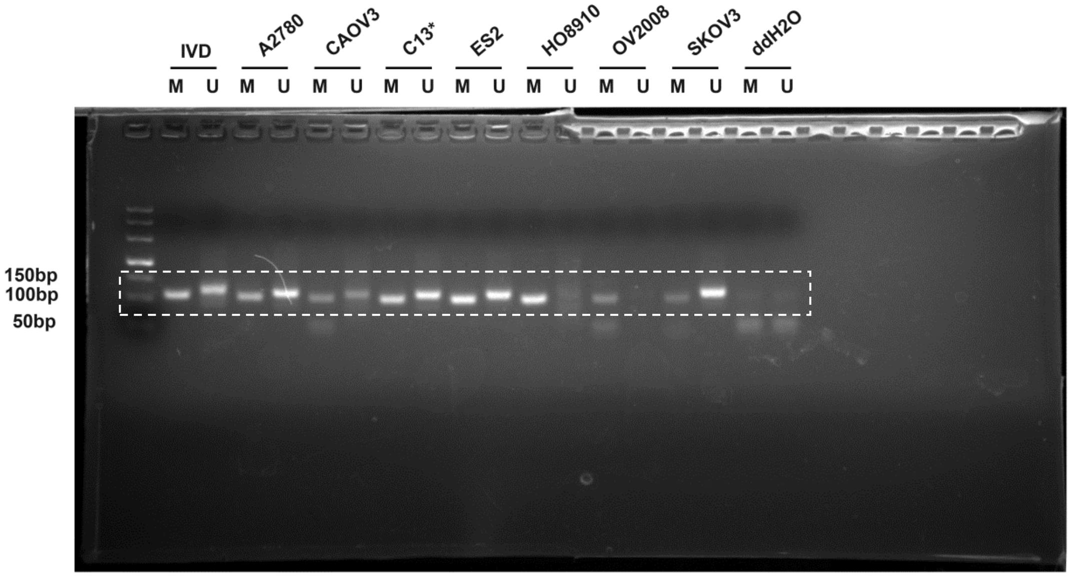

Supplementary Figure S10. Full length gel of Supplemental Figure S4a. White dotted lines show the cropping locations. Brightness was adjusted during processing this gel.

Supplemental Figure S11

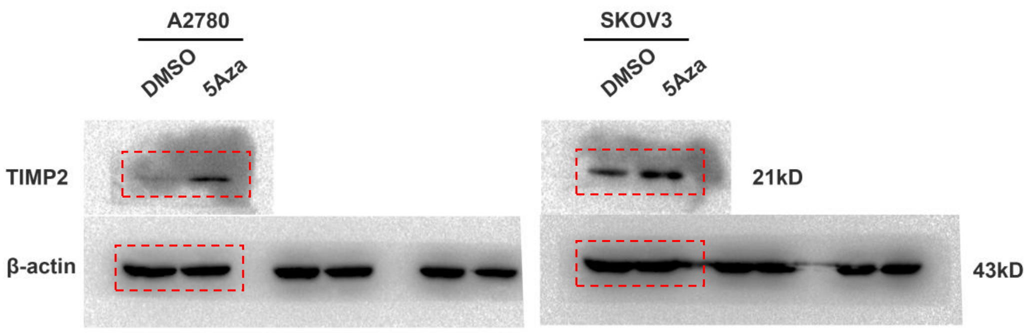

Supplementary Figure S11. Full length blots of Figure 5b. Red dotted lines show the cropping locations.

Supplemental Figure S12

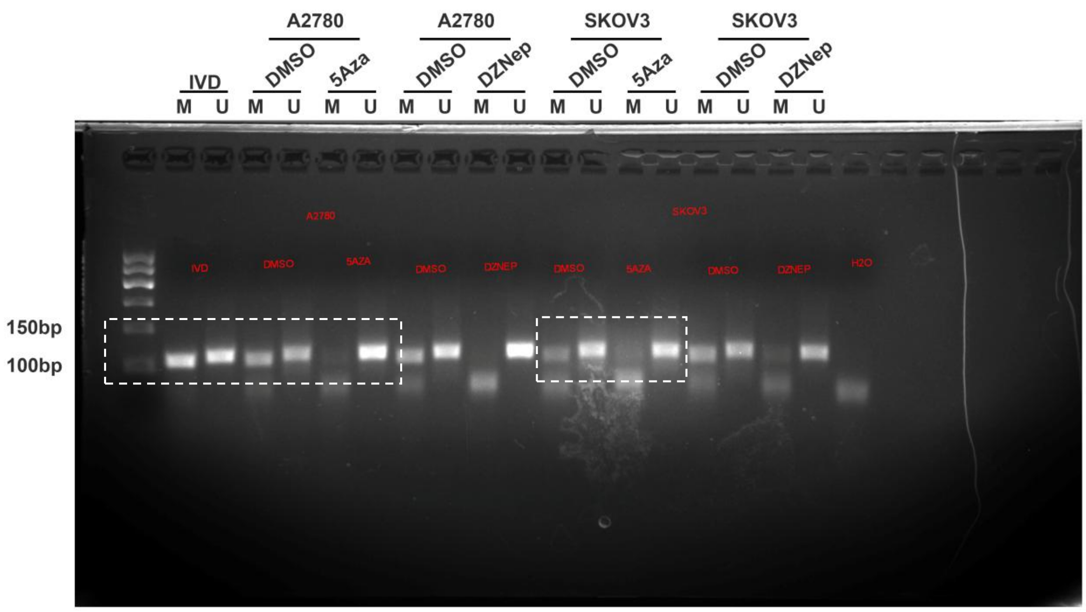

Supplementary Figure S12. Full length gel of Figure 5c. White dotted lines show the cropping locations. Brightness was adjusted during processing this gel.

Supplemental Figure S13

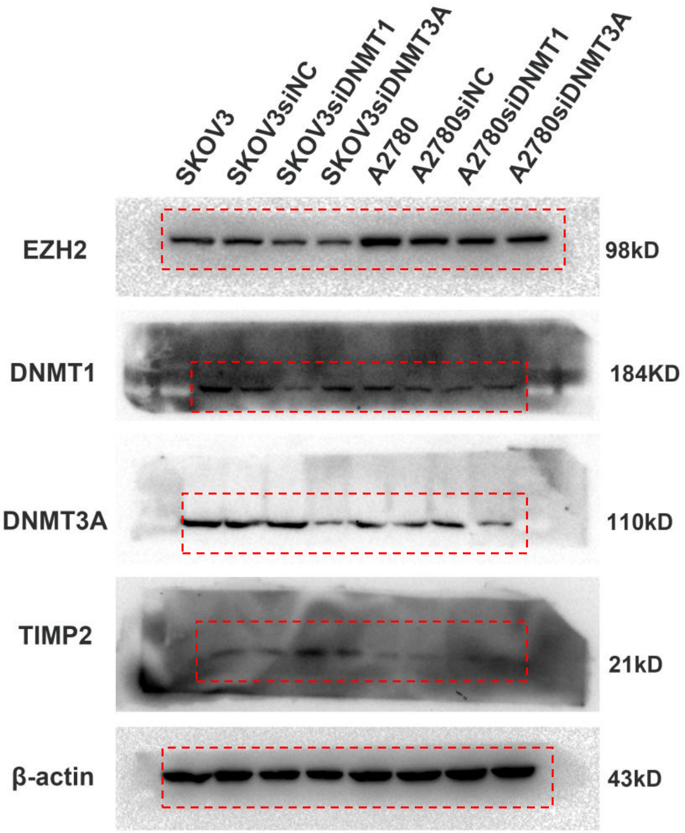

Supplementary Figure S13. Full length blots of Figure 5e. Red dotted lines show the cropping locations. Brightness was adjusted during processing these blots.

Supplemental Figure S14

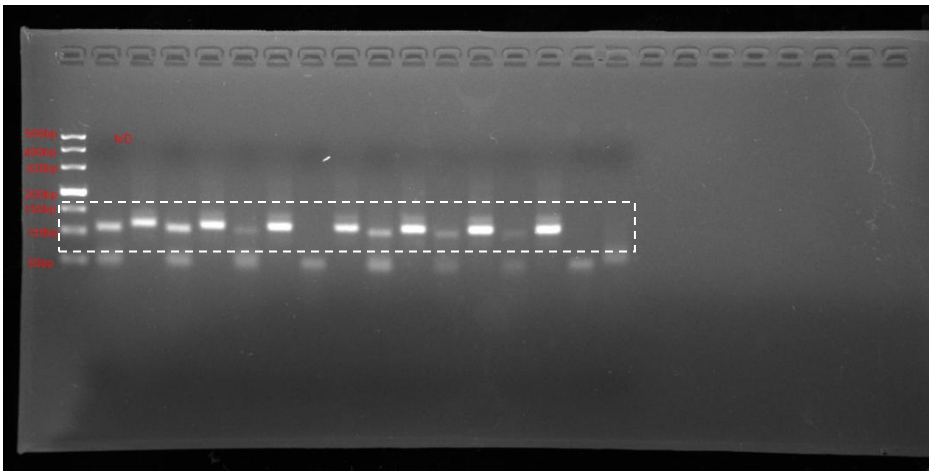

Supplementary Figure S14. Full length blots of Figure 5f. White dotted lines show the cropping locations. Brightness was adjusted during processing this gel.

Supplemental Figure S15

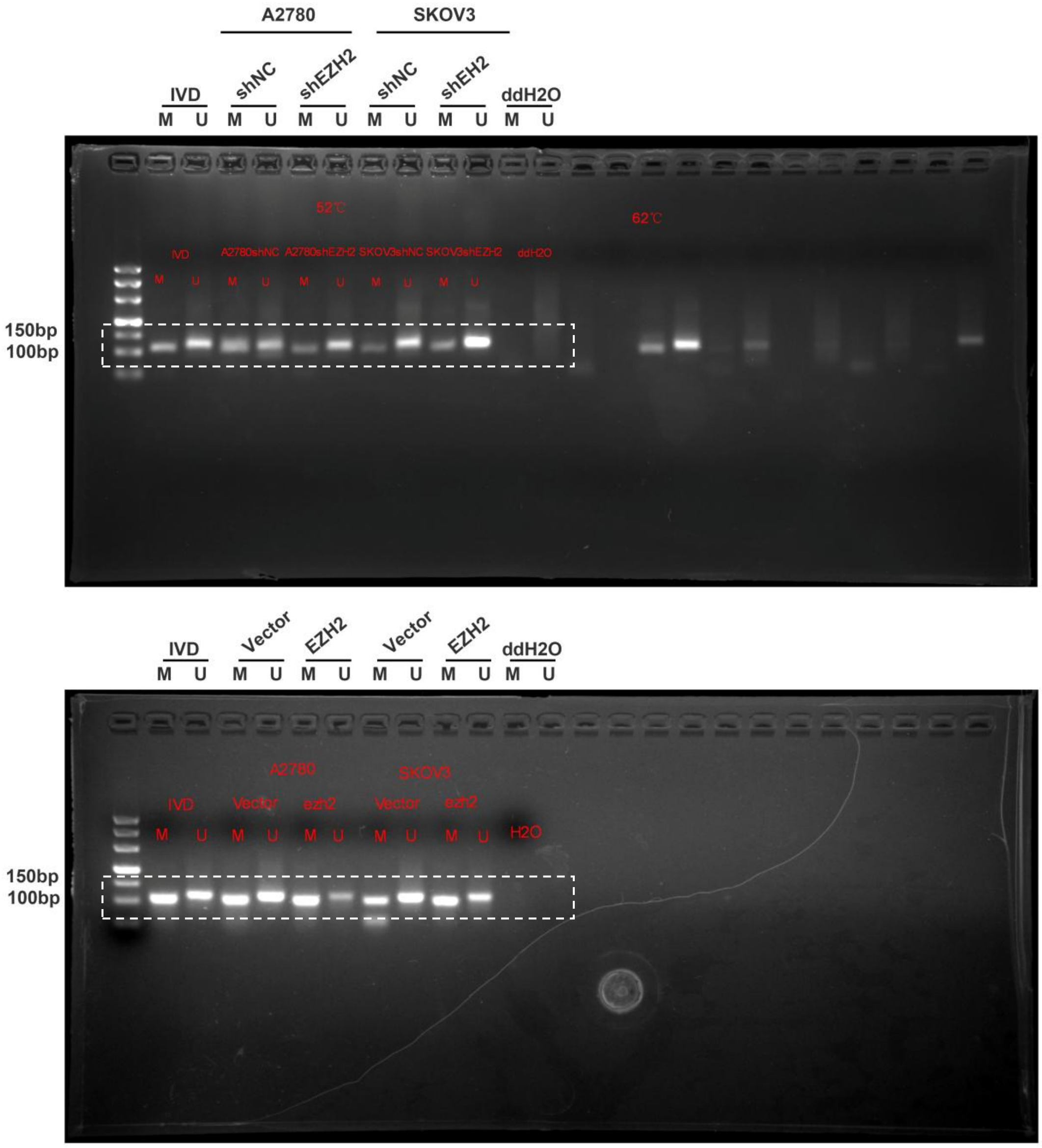

Supplementary Figure S15. Full length gels of Figure 5g. White dotted lines show the cropping locations. Brightness was adjusted during processing these gels.

Supplemental Figure S16

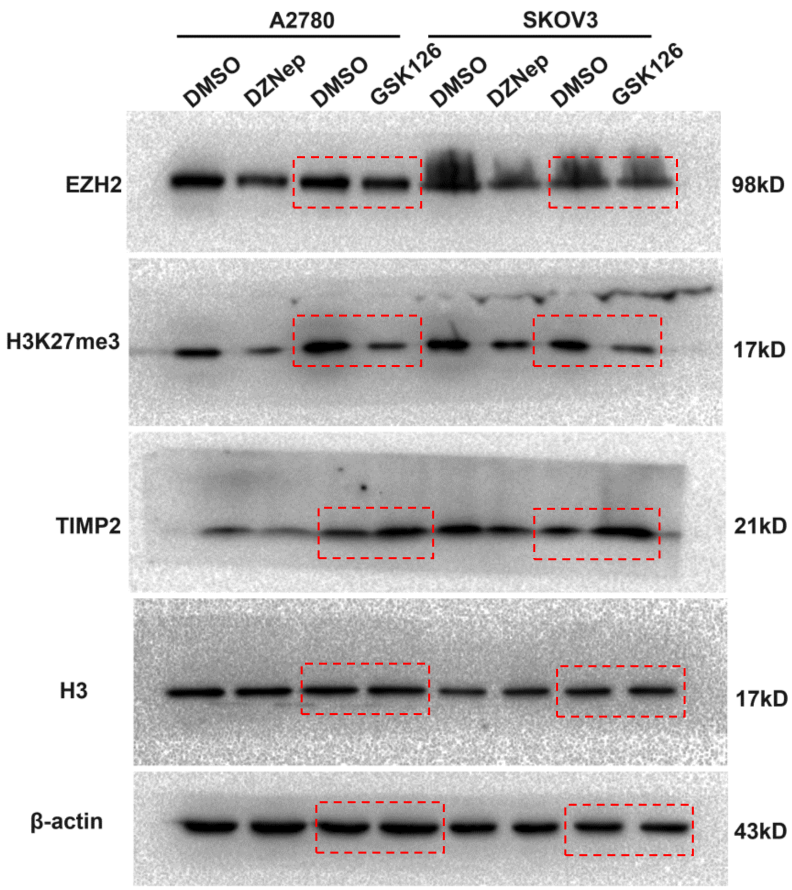

Supplementary Figure S16. Full length blots of Figure 5i. Red dotted lines show the cropping locations.

Supplemental Figure S17

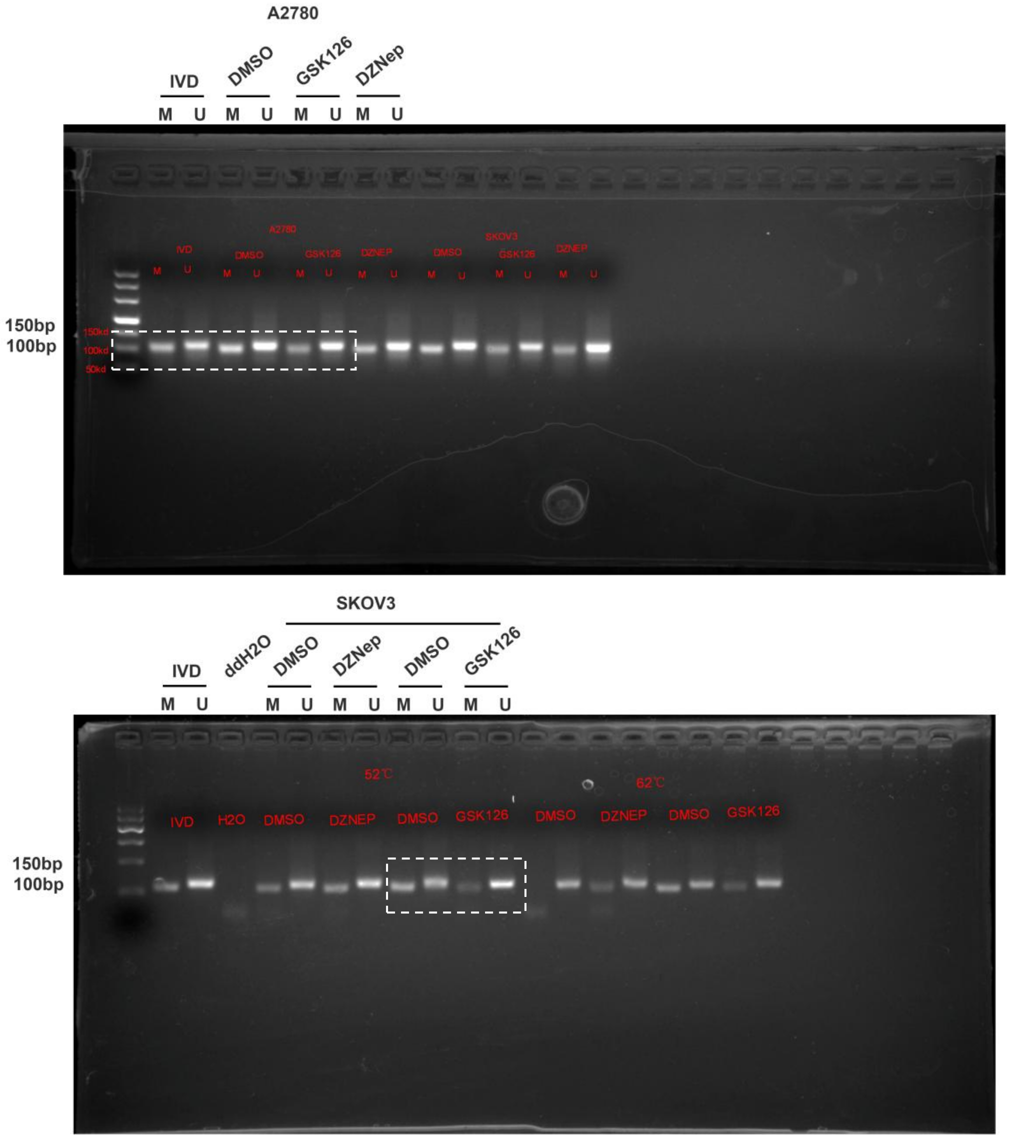

Supplementary Figure S17. Full length gels of Figure 5j. White dotted lines show the cropping locations. Brightness was adjusted during processing these gels.

Supplemental Figure S18

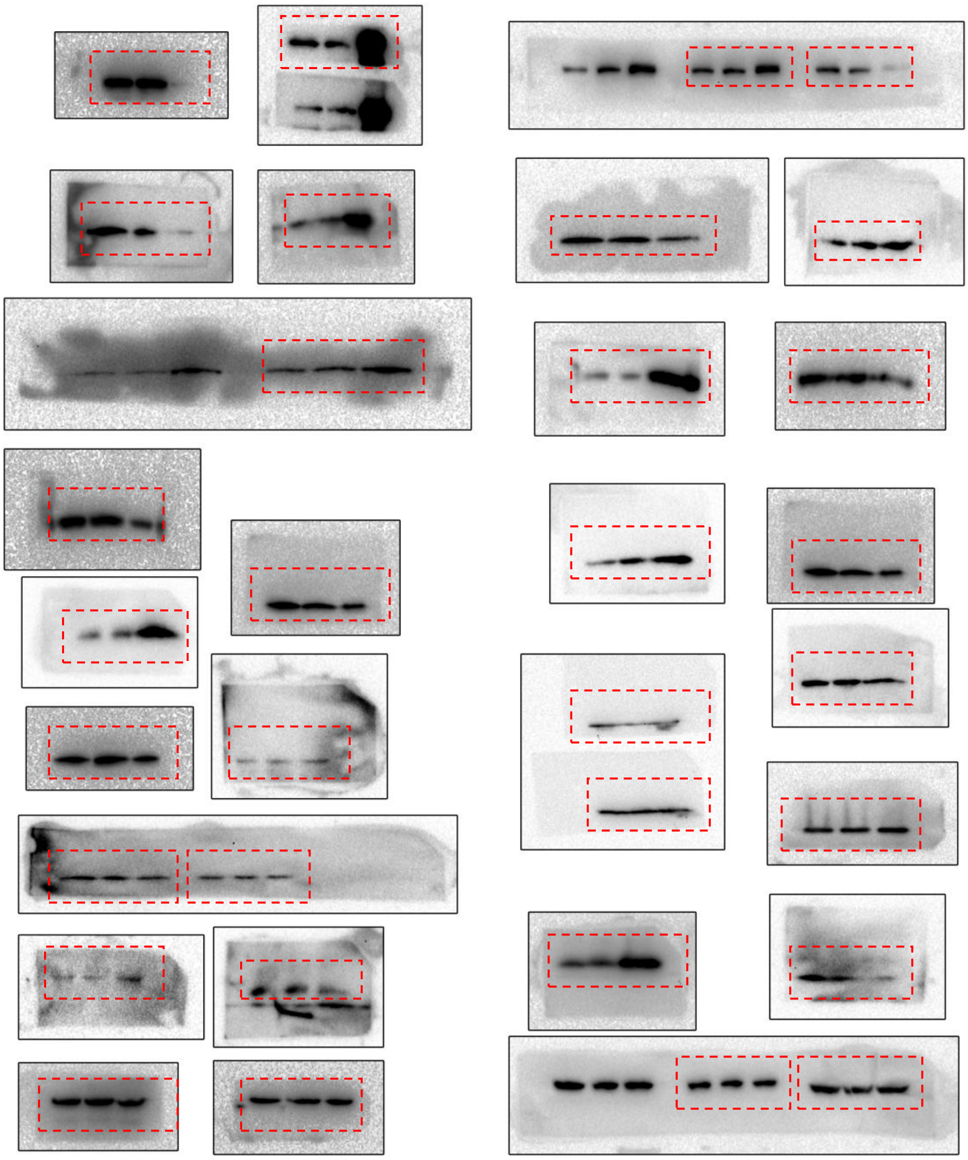

Supplementary Figure S18. Full length blots of Figure 7a. Red dotted lines show the cropping locations. Brightness was adjusted during processing these blots.

Supplemental Figure S19

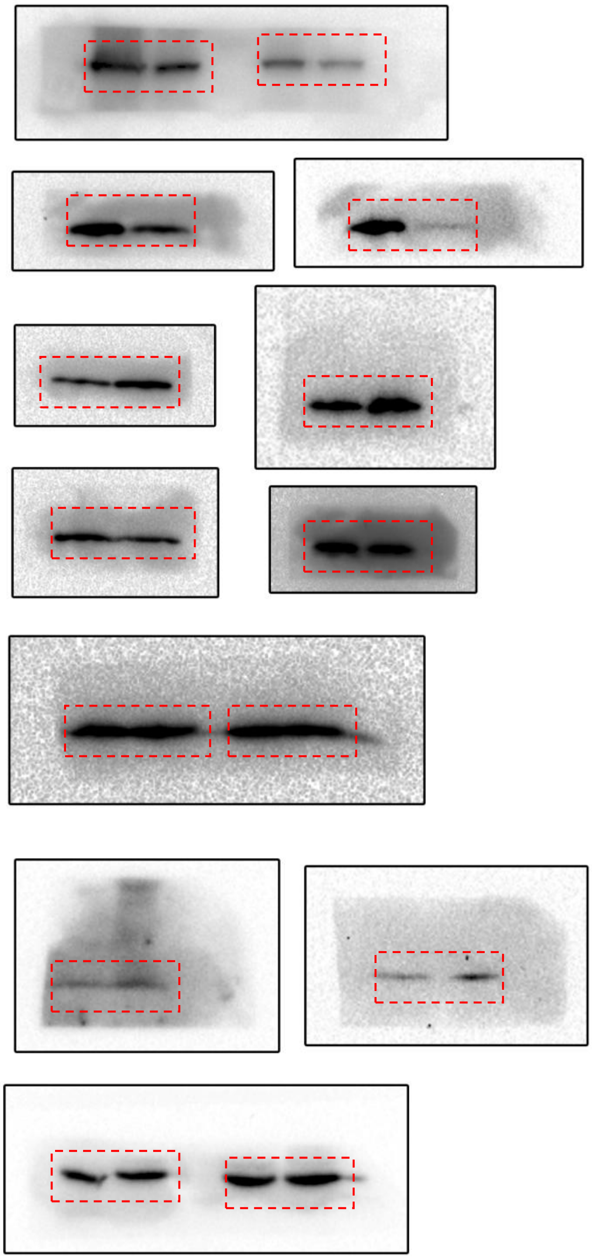

Supplementary Figure S19. Full length blots of Figure 7b. White dotted lines show the cropping locations. Brightness was adjusted during processing these blots.
